# Phototherapy-based combination strategies for bacterial infection treatment

**DOI:** 10.7150/thno.52729

**Published:** 2020-10-30

**Authors:** Guoqing Wei, Guang Yang, Yi Wang, Hezhong Jiang, Yiyong Fu, Guang Yue, Rong Ju

**Affiliations:** 1 Chengdu Women's and Children's Central Hospital, School of Medicine, University of Electronic Science and Technology of China, Chengdu, 611731, PR China.; 2 College of Medicine, Southwest Jiaotong University, Chengdu, 610031, PR China.; 3 School of Life Science and Engineering, Southwest Jiaotong University, Chengdu, 610031, PR China.

**Keywords:** Bacterial infection, Multidrug-resistance, Phototherapy, Combinatorial strategies, Nanomedicine

## Abstract

The development of nanomedicine is expected to provide an innovative direction for addressing challenges associated with multidrug-resistant (MDR) bacteria. In the past decades, although nanotechnology-based phototherapy has been developed for antimicrobial treatment since it rarely causes bacterial resistance, the clinical application of single-mode phototherapy has been limited due to poor tissue penetration of light sources. Therefore, combinatorial strategies are being developed. In this review, we first summarized the current phototherapy agents, which were classified into two functional categories: organic phototherapy agents (*e.g.,* small molecule photosensitizers, small molecule photosensitizer-loaded nanoparticles and polymer-based photosensitizers) and inorganic phototherapy agents (*e.g.,* carbo-based nanomaterials, metal-based nanomaterials, composite nanomaterials and quantum dots). Then the development of emerging phototherapy-based combinatorial strategies, including combination with chemotherapy, combination with chemodynamic therapy, combination with gas therapy, and multiple combination therapy, are presented and future directions are further discussed. The purpose of this review is to highlight the potential of phototherapy to deal with bacterial infections and to propose that the combination therapy strategy is an effective way to solve the challenges of single-mode phototherapy.

## Introduction

Bacterial infections are caused by harmful bacteria invading the host, which may cause severe diseases including pneumonia, tuberculosis, sepsis, cholera, meningitis and osteomyelitis 1-5. Although the discovery of antibiotics has been saving millions of lives 6, unfortunately, conventional antibiotics treatment has problems such as a low utilization rate and severe side effects 7-9. More seriously, the overuse and misuse of antibiotics inevitably increase the prevalence of multidrug-resistant (MDR) pathogenic bacteria 10, which has become a global public health problem in the past decades 6. According to statistics from the World Health Organization (WHO), nearly 80% of MDR microorganisms have arisen due to the global overuse or misuse of antibiotics, and infection by these strains is accompanied by severe adverse effects such as thrombophlebitis and epidermal necrolysis 11. Under this circumstance, massive efforts have been made to develop new strategies for efficient bacterial infections therapy without causing drug resistance.

The use of light in modern medicine was introduced in the 19^th^ century. Based on the advances in our understanding of the physical properties of light and light-matter interactions, medical technology has been developed in parallel 12. A classic example of the early success of light-induced therapy was the treatment of lupus vulgaris with ultraviolet (UV) light, which was discovered by physician Niels Finsen, who won the Nobel Prize in Physiology or Medicine in 1903 for this discovery. A notable milestone in the 1960s was the treatment of severe hyperbilirubinemia using blue-light phototherapy, which subsequently cured millions of infants of this condition 13. Presently, numerous laser-based diagnostic and therapeutic devices have been widely used in clinical practice.

**Figure 1:**
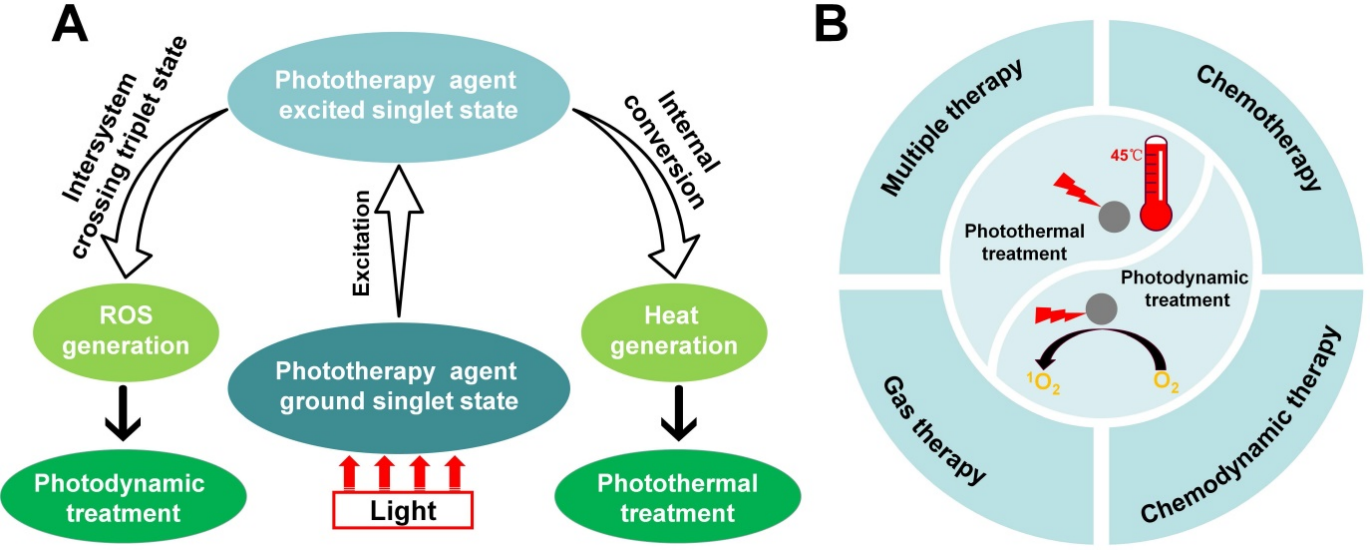
(**A**) The mechanism of photodynamic treatment or photothermal treatment activity by phototherapy agent. (**B**) Combination of different therapies with phototherapy (photodynamic treatment and photothermal treatment), including combination with chemotherapy, combination with chemodynamic therapy, combination with gas therapy and multiple combination therapy.

Over recent decades, the developments of phototherapy antibacterial strategies, such as photodynamic treatment and photothermal treatment 14, have attracted striking attention in nanomedicine because of their good controllability. Different from conventional antibiotics, phototherapy rarely causes bacterial resistance 15. Therefore, phototherapy is a minimally invasive and effective modality that provides a convenient method for ablating bacterial infections using light irradiation in a clinically safe manner 16, 17. **Figure 1A** shows that the mechanism of photodynamic treatment or photothermal treatment activity by a phototherapy agent. Despite the rapid progress and distinctive advantage of phototherapy, single-mode phototherapy techniques face several challenges, including limitations in targeting the infection site. Fortunately, combination with chemotherapy, chemodynamic therapy, gas therapy and multiple combination therapy, can effectively combat these challenges while maximizing the advantage of each therapeutic mode (**Figure 1B**). In this review, we aim to present a discussion on the significant research progress in combinatorial strategies using phototherapy. Firstly, we introduced phototherapeutic agents systematically, summarized the development of phototherapy and discussed the challenges of single-mode phototherapy. Furthermore, the advantages of combination therapy for the treatment of bacterial infections were emphasized. Finally, we discussed the major challenges and problems facing therapeutic nanomedicine in bacteria infectious therapy, and propose possible future directions in this field.

## Phototherapy agents for bacterial infection therapy

Photosensitizers play an important role in phototherapy. The first generation photosensitizers mainly include hematoporphyrin derivatives and porfimer sodium, and their phototherapy performance was limited by the short wavelength of excitation in deep tissues. To solve the disadvantage of the first generation photosensitizers, researchers further developed second generation photosensitizers with near-infrared activation. However, the low cell/tissue specificity of the second generation photosensitizers still prevented them from being used in clinical therapeutics. Over the past few years, novel nanomaterial-based photosensitizers have been received extensive attention and been studied, and these photosensitizers can be divided into organic and inorganic agents. In this part, we will compare the advantages and disadvantages of these two kinds of agents.

### Organic phototherapy agents

Small molecule photosensitizers, small molecule photosensitizers-loaded nanoparticles and polymer-based photosensitizers are the three primary classifications of organic phototherapy agents, all of which have several common advantages, including 1) good biocompatibility; 2) structures and performances that are easy to characterize; 3) easy chemical modification.

#### Small molecule photosensitizers

**Figure 2:**
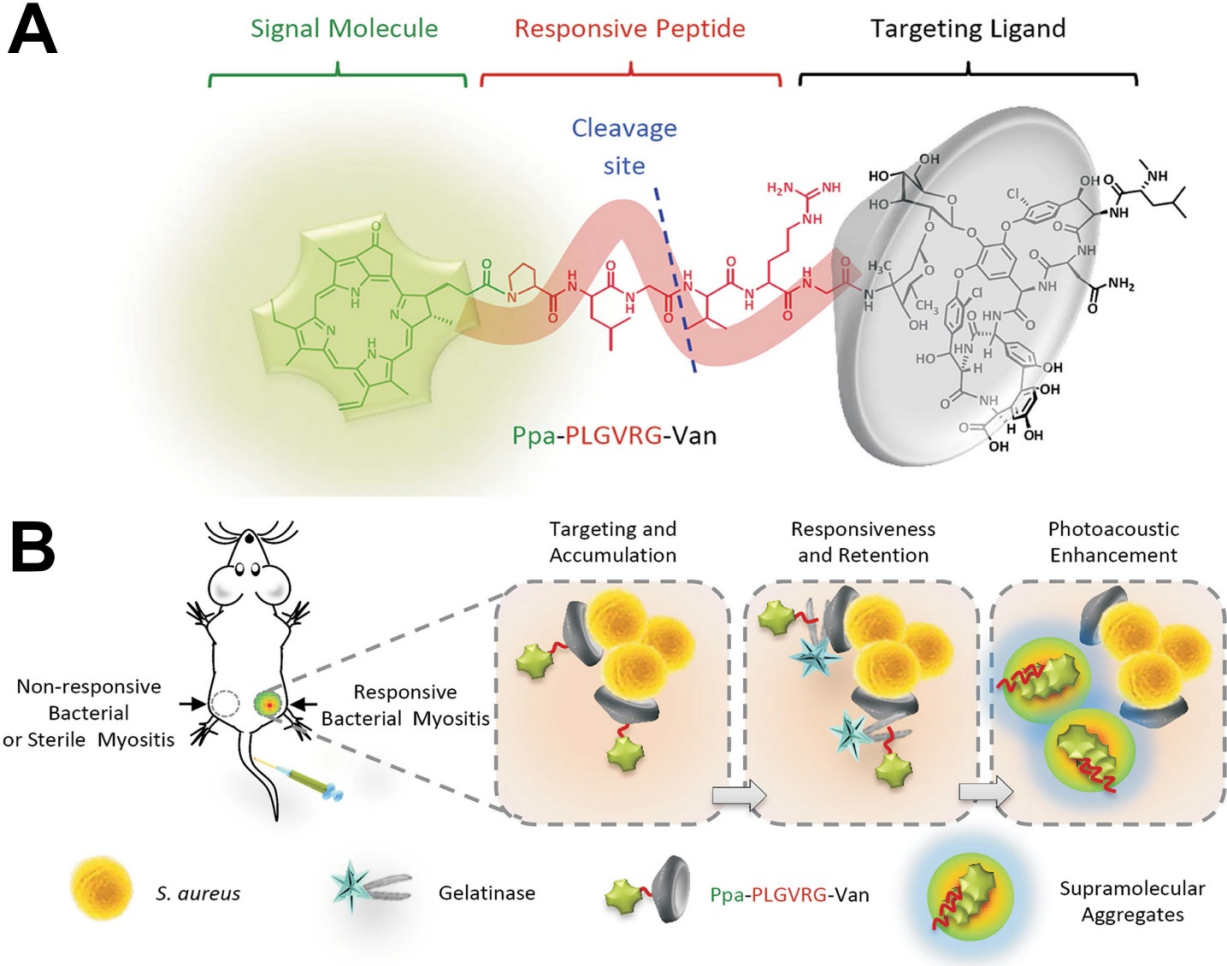
(**A**) Constituent elements of Ppa-PLGVRG-Van. Ppa: pyropheophorbide-α, signal molecule; PLGVRG: Pro-Leu-Gly-Val-Arg-Gly, an enzyme-responsive peptide linker; Van: vancomycin, a targeting ligand. (**B**) Illustration of bacterial infection imaging based on an *in vivo* aggregation strategy^18^. Copyright 2016, Wiley-VCH.

Recently, the small molecules commonly used as organic phototherapy agents mainly including protoporphyrin IX (PpIX), chlorine e6 (Ce6), indocyanine green (ICG) and other photosensitizers. However, several disadvantages still remain in the use of small molecules phototherapy agents. For example, due to the lack of the ability to target bacteria or infection sites, the enrichment of small molecule phototherapy agents at the infected site was not enough, which would further affect their therapeutic performance. To solve this problem, Wang *et al.* 18 developed a new molecular probe (Ppa-PLGVRG-Van), including a signal molecule (pyropheophorbide-α, Ppa), an enzyme-responsive peptide linker (Pro-Leu-Gly-Val-Arg-Gly, PLGVRG), and a targeting ligand (vancomycin, Van) (**Figure 2A**). **Figure 2B** shows that Ppa-PLGVRG-Van could accumulate at the site of responsive bacterial myositis *via* the targeting molecule (Van). Then, the peptide linker is cleaved by gelatinase to make the supramolecular form of hydrophobic building blocks, which readily self-aggregates *in situ* and significantly enhances the photoacoustic signal for detecting the bacterial infection by imaging. Thus, the specific accumulation of Ppa-PLGVRG-Van at the bacterial infection sites leads to a significant function of Ppa *in situ*. We believe that this strategy of chemically functionalizing phototherapy agents can be expanded to enhance the therapeutic effect of other phototherapy agents in bacterial infections.

#### Small molecule photosensitizer-loaded nanoparticles

To enhance the therapeutic effect of small molecule photosensitizers, in addition to the above strategies, another approach is to the extend circulation time of photosensitizers, and enhance the stability at the same time. To achieve this goal, the small molecule photosensitizers can be conjugated with a variety of synthetic biomaterials, or encapsulated into nanoparticles to form small molecule photosensitizer -loaded nanoparticles, such as polymer nanoparticles, liposome, upconversion nanoparticles and other inorganic nanoparticles. All the small molecule photosensitizers -loaded nanoparticles and the corresponding information can be found in **Table 1.** The payload of conjugated or encapsulated drugs can be enriched in the bacterial infection zone *via* the enhanced penetration and retention (EPR) effect 38-40, and kill bacteria under light irradiation. Moreover, to reduce the side effects, the components of nanoparticles are also required to be degradable *in vivo* to guarantee rapid clearance after eradicating bacterial infections 41. Therefore, biodegradability is one of the important considerations in developing small molecule photosensitizer-loaded nanoparticles. When bacteria infects and invades the host, the expression of enzymes (*e.g.,* phosphatase, phospholipase and protease) at the site of infection will significantly increase, the pH value will decrease to an acidic pH, and the local temperature will increase, thus forming a special microenvironment of bacterial infection 40, 42, 43. Therefore, the prepared small molecule photosensitizer-loaded nanoparticles, which can respond to the microenvironment of bacterial infected tissues, are the best choices to improve the phototherapy efficacy and helpful to slow down the emergence of side effects. For example, Yan *et al.* 24 synthesized silica nanoparticles doped with Ce6. This hybrid structure exhibits enhanced photostability and a high antibacterial efficiency towards *Staphylococcus aureus* (*S. aureus*) and *methicillin-resistant S. aureus* (MRSA). Therefore, this work demonstrates an effective platform to improve the efficiency of small molecule photosensitizers for better treatment of wound infections.

In addition, nanosized metal organic frameworks (NMOFs) exhibit an advantage of encapsulating many small molecules due to their highly porous structure. In addition, they can reduce the self-quenching of small molecule photosensitizers and show great photodynamic treatment efficacy. For example, Bagchi *et al.* 30 prepared a squaraine (SQ)-encapsulated zeolitic imidazolate framework-8 (ZIF-8) NMOF (ZIF8-SQ) by a postsynthetic strategy. The insertion of SQ within the ZIF-8 cavity could lower the free diffusion of SQ molecules in biological media and limit the SQ aggregation possibilities. *In vitro* antibacterial tests confirmed that ZIF8-SQ could provide unparalleled photodynamic treatment action against MRSA.

In recent years, bioresponsive nanoparticles have achieved remarkable results in the treatment of bacterial infections *via* phototherapy 44, 45. Sun *et al.* developed pH sensitive nanoparticles combined with ammonium methylbenzene blue for the photodynamic treatment of bacterial infections, showing an efficient biofilm eradication capability 31. Wang *et al.* 44 designed an antibacterial hydrogel in an acidic environment. The hydrogel loading photothermal agent could cure wounds infected with a MRSA biofilm in a short period of time.

**Figure 3:**
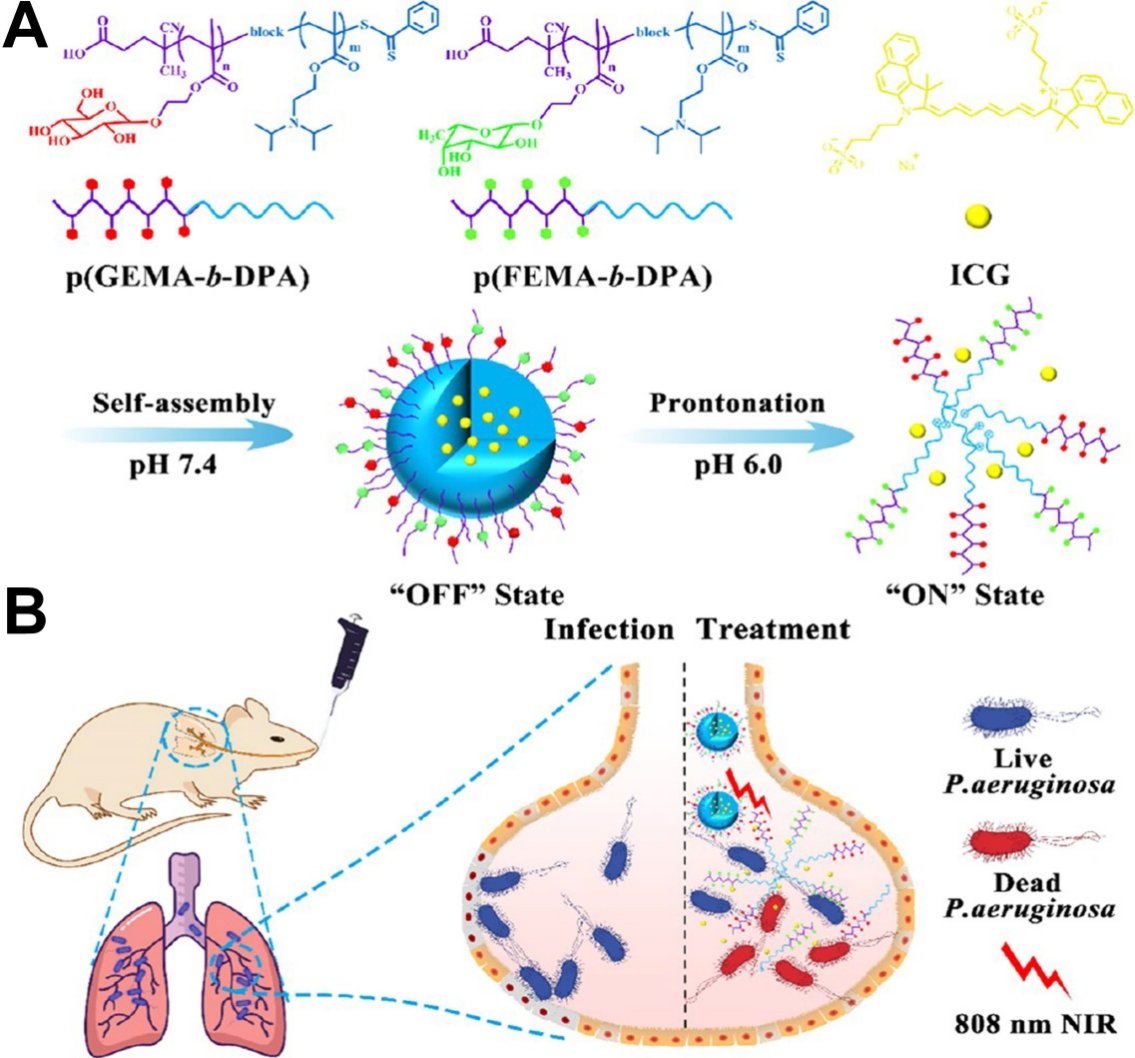
(**A**) Schematic illustration of preparation of the nanovehicle (gadofullerene nanoparticles, GFNPs) and (**B**) killing* P. aeruginosa* at infected alveoli by photothermal treatment 22. Copyright 2019, ACS Publications.

To realize the targeted delivery of photosensitizers, target ligands can be modified on nanoparticles. For example, Zhang *et al.* 22 prepared a pH-tunable ON/OFF nanovehicle with a heteromultivalent and glycomimetic shell for targeting bacterial lectins and an acid-responsive core for the controlled release of phototherapeutic agent at infectious sites. As shown in **Figure 3**, the shell of the nanovehicle was composed of heteromultivalent glycomimetics, which could bind tightly to bacteria and subsequently inhibit biofilm formation. The core was composed of acid-sensitive polymers that changed from hydrophobic to hydrophilic through protonated effects under an acidic microenvironment. Under physiological conditions, the hydrophobic core of nanotherapeutics was in the “OFF” state and locks the phototherapeutic agent in the cage. Upon arriving at the infection site, the nanotherapeutics were in the “ON” state under an acid microenvironment, promoting the release of phototherapeutic agent which induced oxygen species (ROS) and heat under NIR laser illumination. Therefore, this heteromultivalent ligand-decorated nanovehicle could effectively avoid side effects on normal tissue and the occurrence of bacterial resistance.

**Figure 4:**
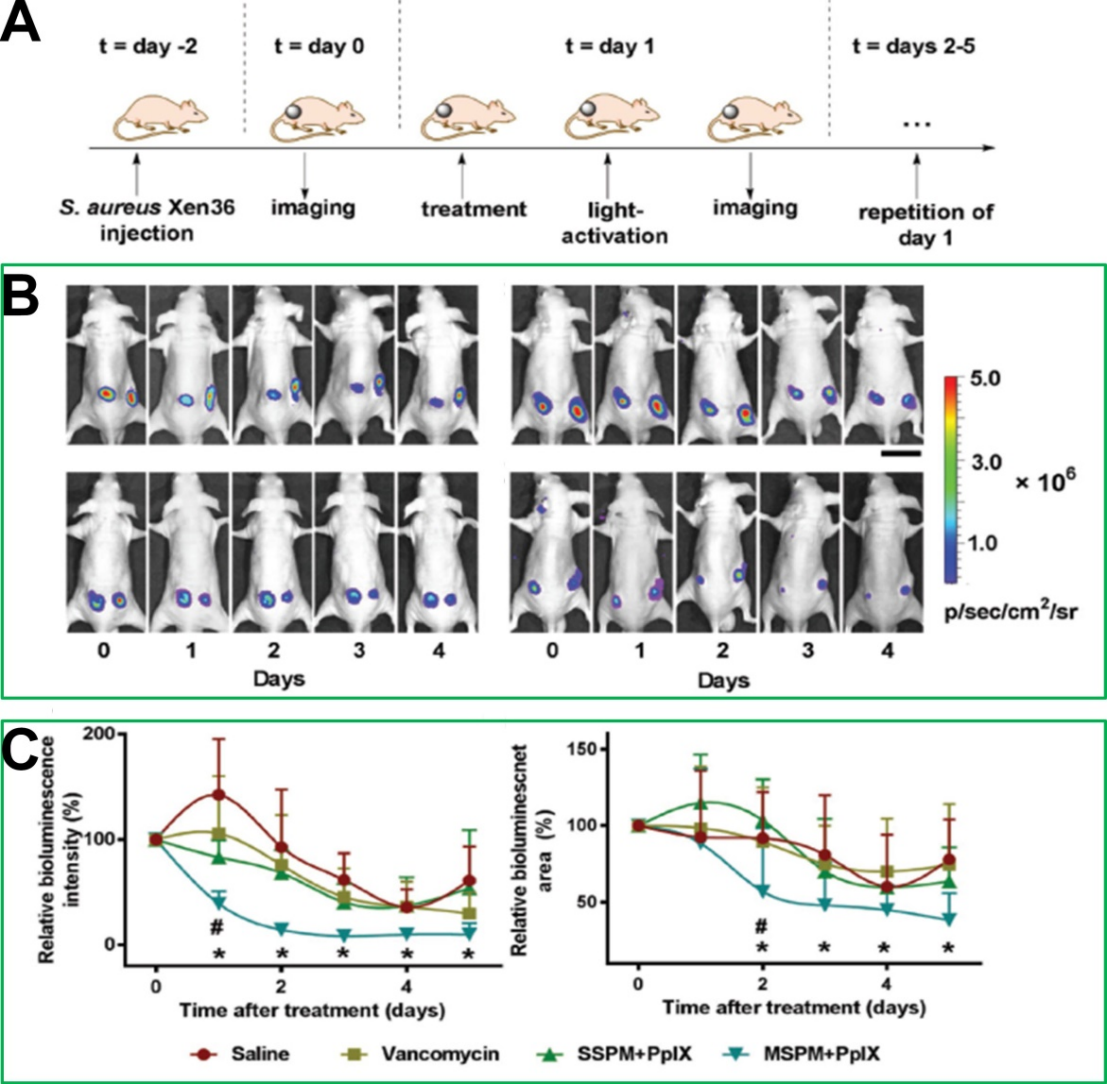
(**A**) The building process of the vancomycin-resistant staphylococcal infection in a murine model and the photodynamic treatment procedure. (**B**) Bioluminescence images after the treatments with different strategies. (**C**) Relative bioluminescence intensities and relative bioluminescence areas after each treatment 23. Copyright 2017, Wiley-VCH.

On the other hand, the targeting of nanoparticles to bacterial cells can be promoted *via* electrostatic interaction. For example, Shi *et al.* 23 designed and synthesized pH-sensitive mixed-shell-polymeric-micelles (MSPMs) composed of poly(ethylene glycol)-*b*-poly(ε-caprolactone) (PEG-*b*-PCL) and poly(ε-caprolactone)-*b*-poly(β-amino ester) (PCL-*b*-PAE). The potential of MSPMs could change from a negative charge to a positive charge due to charge-switching of the PAE blocks at a low pH, which would promote the targeting of MSPMs to bacterial cells *via* electrostatic interactions. In a murine model, **Figure 4** shows that the experimental group administered PpIX-loaded MSPMs exhibited a faster recovery rate after infection with vancomycin-resistant *staphylococcal*, while the differences in the bacterial eradication efficacies of the groups treated with saline, vancomycin, and PpIX-loaded single shell-polymeric-micelles (SSPMs) composed of PEG-*b*-PCL were minimal. Taken together, these studies provided promising approaches for the treatment of MDR bacterial infection.

**Figure 5:**
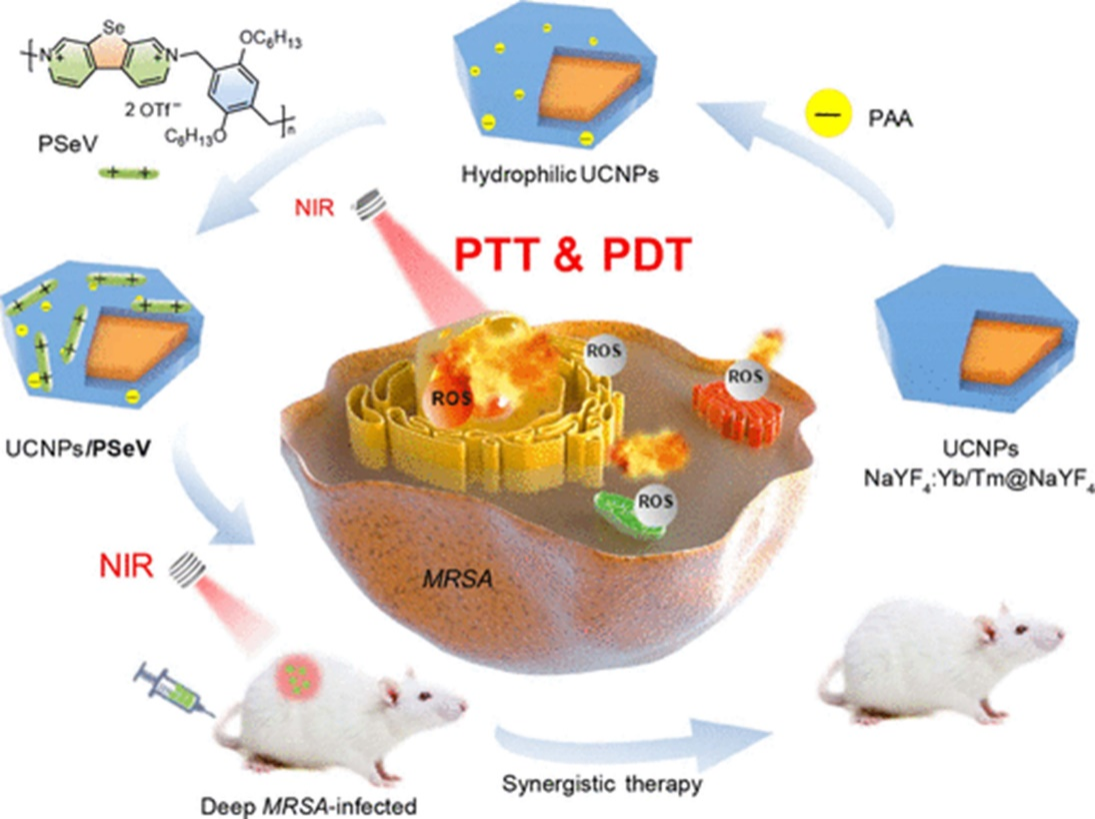
Schematic illustration of the main synthetic procedure and coordinated antimicrobial strategy of photodynamic treatment and photothermal treatment 36. Copyright 2020, ACS Publications.

Because upconversion nanoparticles (UCNPs) have the unique ability of upconverting NIR light to UV and visible emissions 46, they are also commonly used in phototherapy. Sun *et al.* 33 designed and synthesized antibacterial a core-shell UCNPs@SiO_2_(MB), in which the core was NaYF4:Yb,Er,Gd and the shell was methylene blue (MB)-loaded silica, which could convert NIR light into visible photons to activate MB through fluorescence resonance energy transfer (FRET) and generate ROS to rapidly kill both Gram-positive *S. aureus* (94.5%) and Gram-negative *E. coli* (93.2%). To develop highly effective antibacterial UCNPs, Zhou *et al.* 36 reported a photosensitizer (PS) prepared through the self-assembly of poly(selenoviologen) on the surface of core-shell NaYF4:Yb/Tm@NaYF4 UCNPs. **Figure 5** shows a schematic illustration of the main synthetic procedure and synergistic photodynamic treatment and photothermal treatment strategies. The hybrid UCNP/PSeV PS showed a strong ROS generation ability and high photothermal conversion efficiency (∼52.5%) under the mildest reported-to-date irradiation conditions (λ=980 nm, 150 mW/cm^2^, 4 min) and led to a high efficiency in killing MRSA both *in vitro* and* in vivo*.

#### Polymer-based photosensitizers

Polymer-based photosensitizers are in-between small molecules and nanoparticles. Many conjugated polymers and conjugated polymer nanoparticles (CPNs) have been utilized as photodynamic and photothermal treatment agents for bacterial infection therapy 47-51.

For example, Feng *et al.* 51 reported photothermal-responsive CPNs for the rapid and effective killing of bacteria. These CPNs are composed of poly[2,6'-4,8-di(5-ethylhexylthienyl)benzo[1,2-b;3,4-b]dithiophenealt-5-dibutyloctyl-3,6-bis(5-bromothiophen-2-yl)pyrrolo[3,4-c]pyrrole-1,4-dione] (PDPP-DBT) and matrix polymer DSPE-PEG2000-MAL. To enhance the interaction of CPNs with bacteria cells, the resulting CPNs were further modified with the positively charged Tat peptide (RKKRRQRRRC) by chemical conjugation between the maleimide groups and thiol groups. *In vivo* and *in vitro* experimental results confirmed that CPNs-Tat could enhance the interaction with bacteria cells with the formation of CPN-Tat/bacteria aggregates, and these photothermal-responsive CPNs converts light into heat and produces local hyperthermia to kill bacteria within a few minutes under NIR irradiation. Therefore, this photothermal-responsive strategy offers another rapid and effective modality for combating bacterial infections.

### Inorganic phototherapy agents

Compared with organic phototherapy agents, many inorganic phototherapy agents exhibit strong optical absorption and efficient photothermal conversion, and therefore, they are commonly used as photothermal treatment agents (PTTs) to fight bacterial infections. There are many factors influencing the phototherapy performance of inorganic PTTs, including their size, shape, concentration and surface modification. According to the morphology of inorganic PTTs, they can be classified into nanorods, nanocages, nanodots, nanocubes, nanosheets, nanostars, nanoflowers, nanoeggs, nanopopcorn, and numerous other 2D materials 52. In this part, we classify most PTTs into 4 categories: carbon-based nanomaterials (*e.g.,* graphene derivatives and carbon nanotubes), metal-based nanomaterials (*e.g.,* Au and CuS) 53, composite nanomaterials and other nanomaterials (*e.g.,* quantum dots). In fact, overlaps cannot be avoided in some cases for our classification.

#### Carbon-based nanomaterials

Carbon-based nanomaterials have been widely as phototherapy agents studied in antibacterial therapy because of their relatively high bacterial toxicity, negligible mammalian cytotoxicity and other favourable properties. In recent years, graphene-based nanomaterials (GBNs), such as graphene, reduced graphene oxide (rGO), grapheneoxide (GO) and other chemically modified-graphene, have been widely used in antibacterial therapy research. **Table 2** shows the carbon-based nanomaterials commonly used in antibacterial therapy.

**Figure 6:**
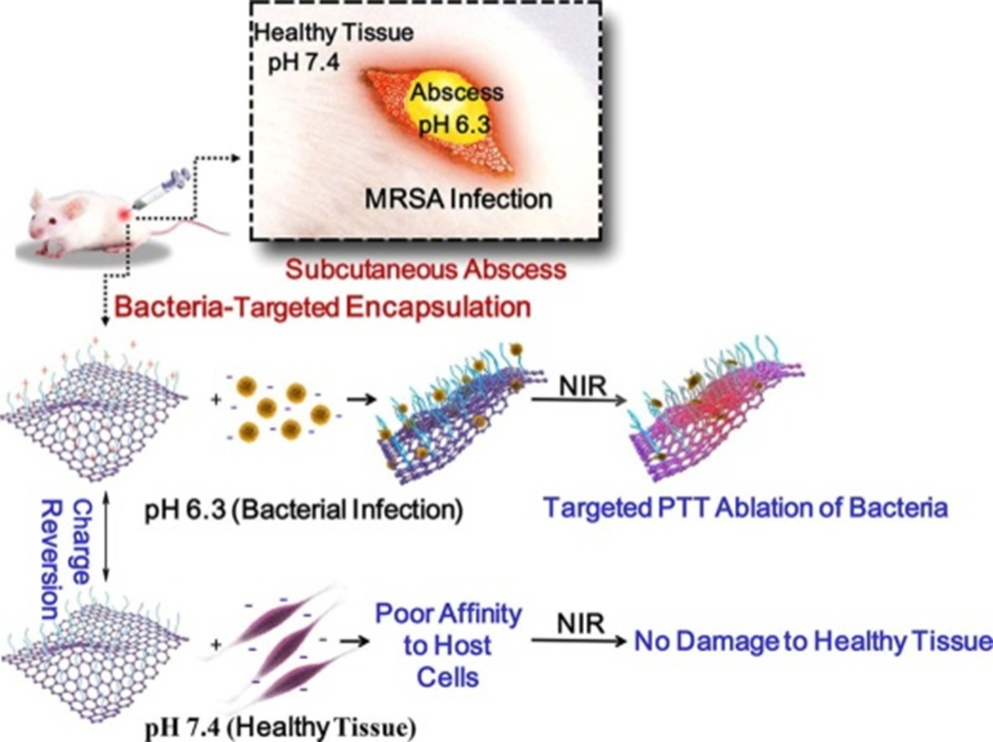
Schematic illustration of the antibacterial mechanism of glycol chitosan conjugated carboxyl graphene (GCS-CG) 54. Copyright 2018, Elsevier.

To achieve the more specific and effective photothermal sterilization of GBNs, they were usually modified with some functional molecules or nanostructures. For example, Qian *et al.* 54 developed surface-adaptive and biocompatible glycol chitosan-conjugated carboxyl graphene (GCS-CG), which could target to the acidic environments of infected abscesses without damage to healthy tissue (**Figure 6**). The* in vitro* experimental results demonstrated that the aqueous solution of GCS-CG showed the rapid production of heat energy with NIR irradiation. This kind of GO-based nanomaterial could be applied as a new potential photothermal treatment agent in treating bacterial infection and even MDR bacteria.

#### Metal-based nanomaterials

**Figure 7:**
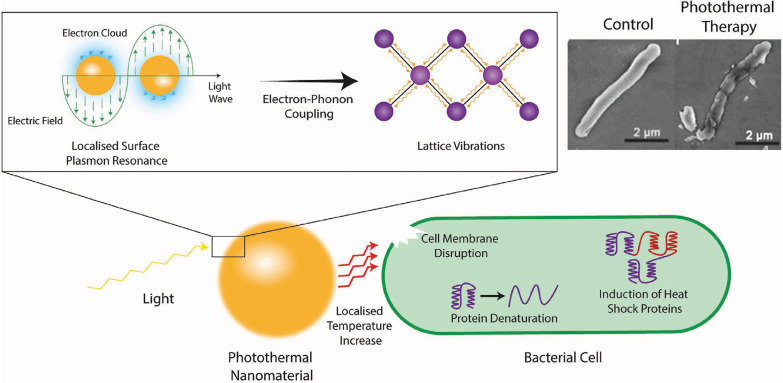
Schematic representation of the photothermal conversion of light to heat and the subsequent antimicrobial mechanism taking place. Top right are scanning electron micrographs of *E. coli* cells before (left) and after (right) treatment with photothermal nanomaterials 69. Copyright 2018, Wiley-VCH.

Metal nanomaterials are the material most commonly used for photothermal treatment due to their broad spectral absorption range and light stability 53, 55. **Figure 7** shows the antibacterial mechanism of the photothermal conversion of metal nanomaterials. In short, the electromagnetic field causes electrons in the conduction band at the surface of the metal nanomaterials to rapidly oscillate 64. This absorbed energy induces vibrations in the metallic lattice *via* electron-photon coupling, which is subsequently transferred into thermal energy causing a local temperature increase 65. In order to kill pathogens specifically, these nanomaterials could target the pathogen by conjugating targeting ligands (such as antibodies) on their surface, which would increase the local temperature around the pathogen and cause cell death through a suite of actions including the denaturation of essential proteins/enzymes, induction of heat shock proteins, disruption of metabolic signaling and rupture of the cell membrane 54, 66-68. The commonly investigated inorganic PTTs and their images with corresponding shape are shown in **Table 3.**

**Figure 8:**
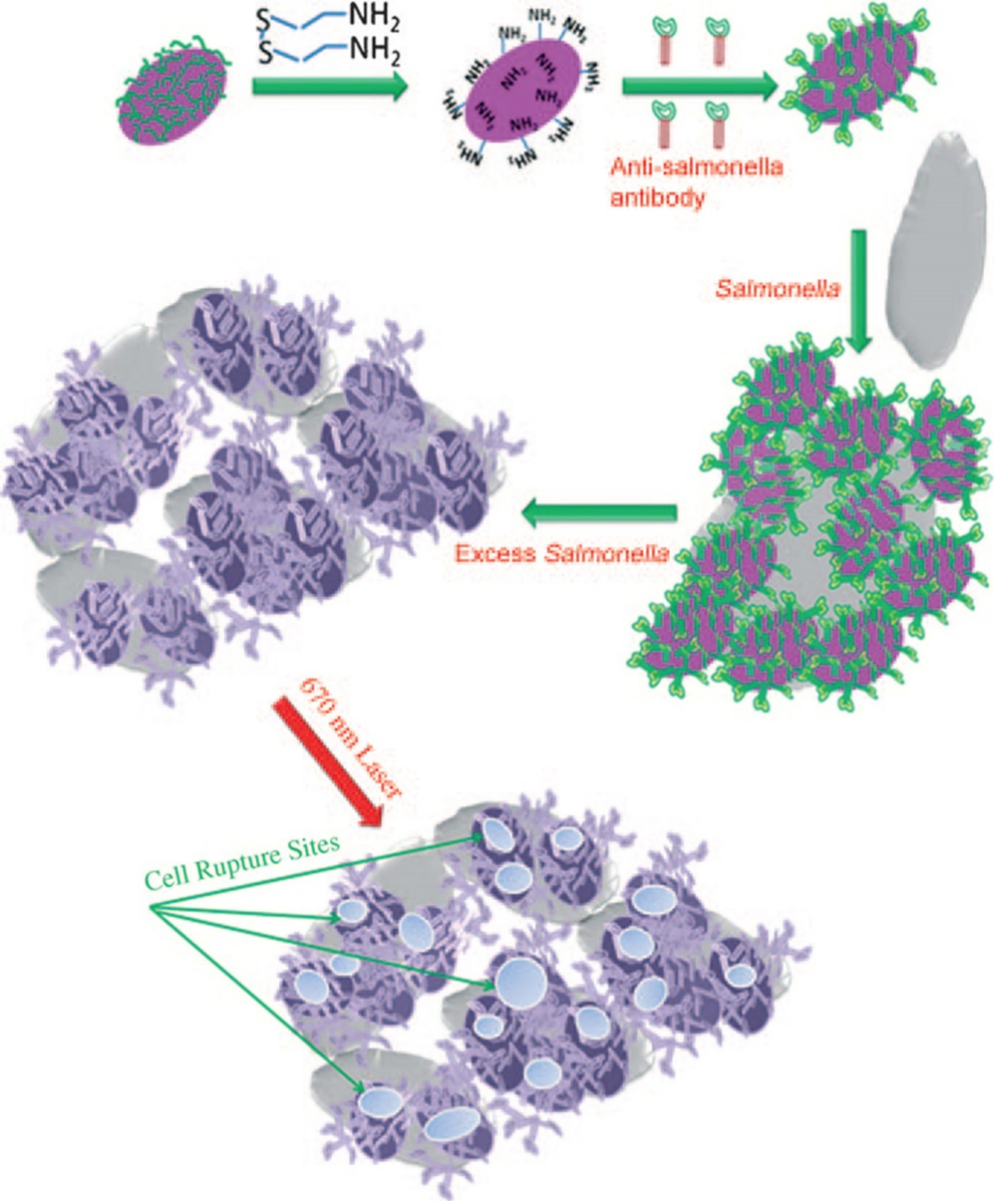
Schematic illustration of antibody- conjugated oval-shaped gold nanoparticles to selectively target and destroy pathogenic bacteria 109. Copyright 2010, Wiley-VCH.

For example, Norman *et al.* 71 prepared gold nanorods with covalently conjugating primary resistance antibodies, which could selectively target the Gram-negative pathogen, *Pseudomonas aeruginosa*. The subsequent attachment of these gold nanorods to the pathogenic bacterial cell surface could significantly reduce the bacteria cell viability under near-infrared radiation (λ=785 nm). Similarly, Wang *et al.* 109 prepared oval-shaped gold nanoparticles, with anti-salmonella antibodies bound on their surface (**Figure 8**). When oval-shaped gold nanoparticles are attached to bacterial cells, the heat locally generated during NIR irradiation (λ=785 nm) could cause irreparable cellular damage of *Salmonella typhimurium* bacteria. The successful bioconjugation of photothermal nanomaterials with targeting ligands shows promise in improving the selectivity to enhance the photothermal treatment effect of metal nanomaterial-based PTTs.

#### Composite nanomaterials

**Figure 9:**
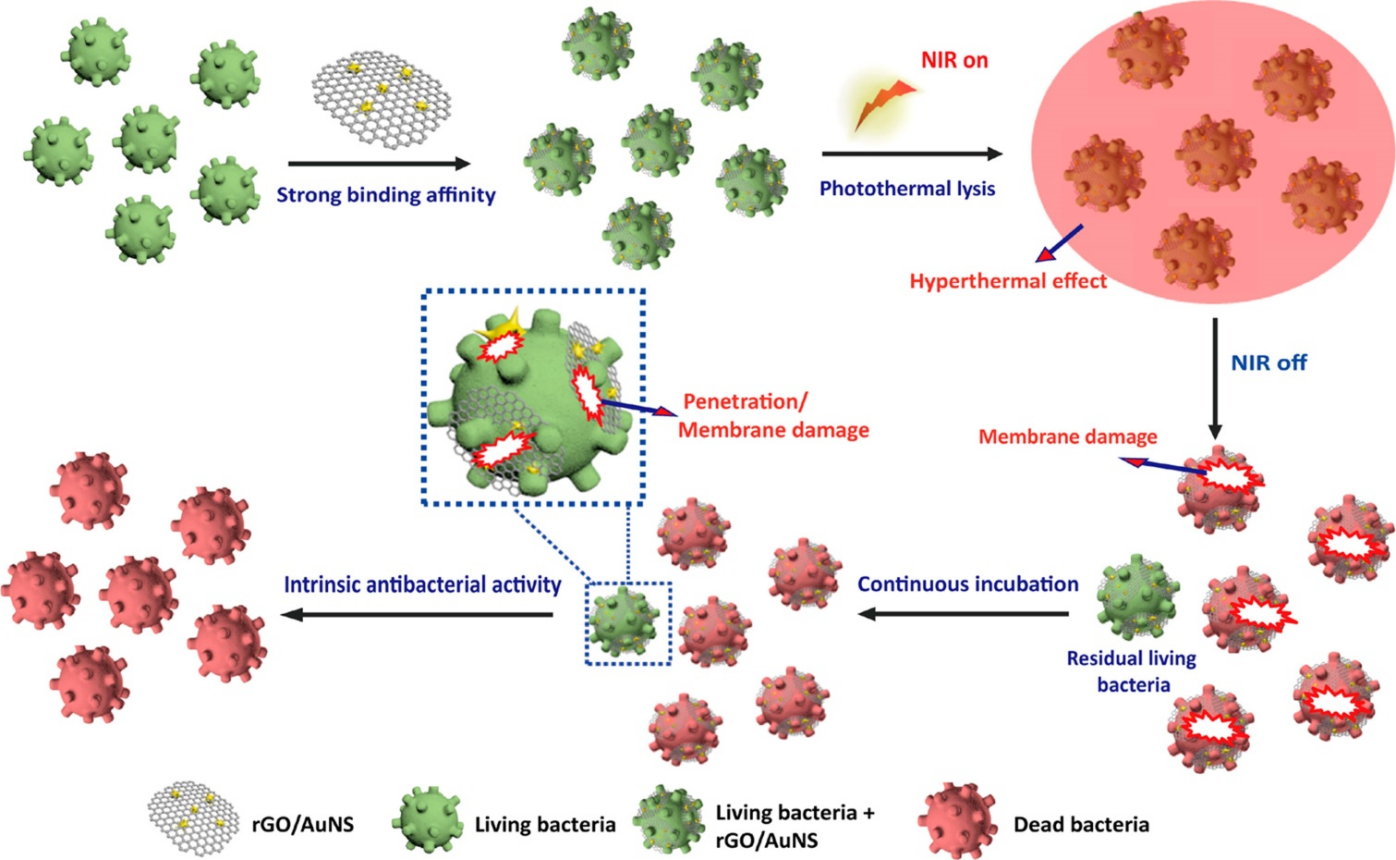
Schematic illustration of 2D reduced graphene oxide supported Au nanostar nanocomposite (rGO/AuNS) triggered antibacterial photothermal lysis 111. Copyright 2019, ACS publication.

To fully exploit the effect of different types of photothermal agents, increasing numbers of composite-based nanomaterials photothermal agents have been designed and prepared 110. For example, Feng *et al.* 111 prepared a rGO and AuNS nanocomposite (rGO/AuNS) *via* a seed-mediated growth method (**Figure 9**). The results of *in vitro* experiments confirmed that MRSA could be killed completely when incubated with rGO/AuNS (50 μg/mL) under NIR irradiation (808 nm, 3 W/cm^2^) for 6 min. Therefore, this indicates that rGO/AuNS can be used as a dual functional photothermal agent for synergistically killing MDR bacteria. Similarly, Luo *et al.* 112 developed a facile assembly of Au NPs decorated GO/nanocellulose paper, which has an intense antimicrobial activity against both Gram-positive and Gram-negative bacteria due to its excellent photothermal conversion performance. Besides, the satisfactory *in vitro* antibacterial performance demonstrated that these functionalized papers could be applied for skin disinfection or medical device sterilization.

In addition, black phosphorus (BP) nanosheets, a rising star among 2D materials, have recently attracted widespread attention in antibacterial applications owing to their high photothermal conversion efficiency, large surface area-to-volume ratio, excellent biocompatibility, and good biodegradation 126. In particular, a variety of composite materials based on black phosphorus and metal nanoparticles have been developed and proved to exhibit an excellent antibacterial efficiency 127, 128. For instance, Aksoy *et al.* 129 designed and prepared new a BP/Au nanocomposite as a photothermal antibacterial agent to kill an important nosocomial pathogen *E. faecalis*. *In vitro* studies have shown that because the BP/Au nanocomposite has higher photothermal conversion efficiency, it more efficiently destroyed the bacterial cell membrane than bare BP, with a biofilm inhibition rate of 58% achieved under NIR light irradiation. The photothermal composite nanomaterials can be found in **Table 4.**

##### Quantum dots

**Figure 10:**
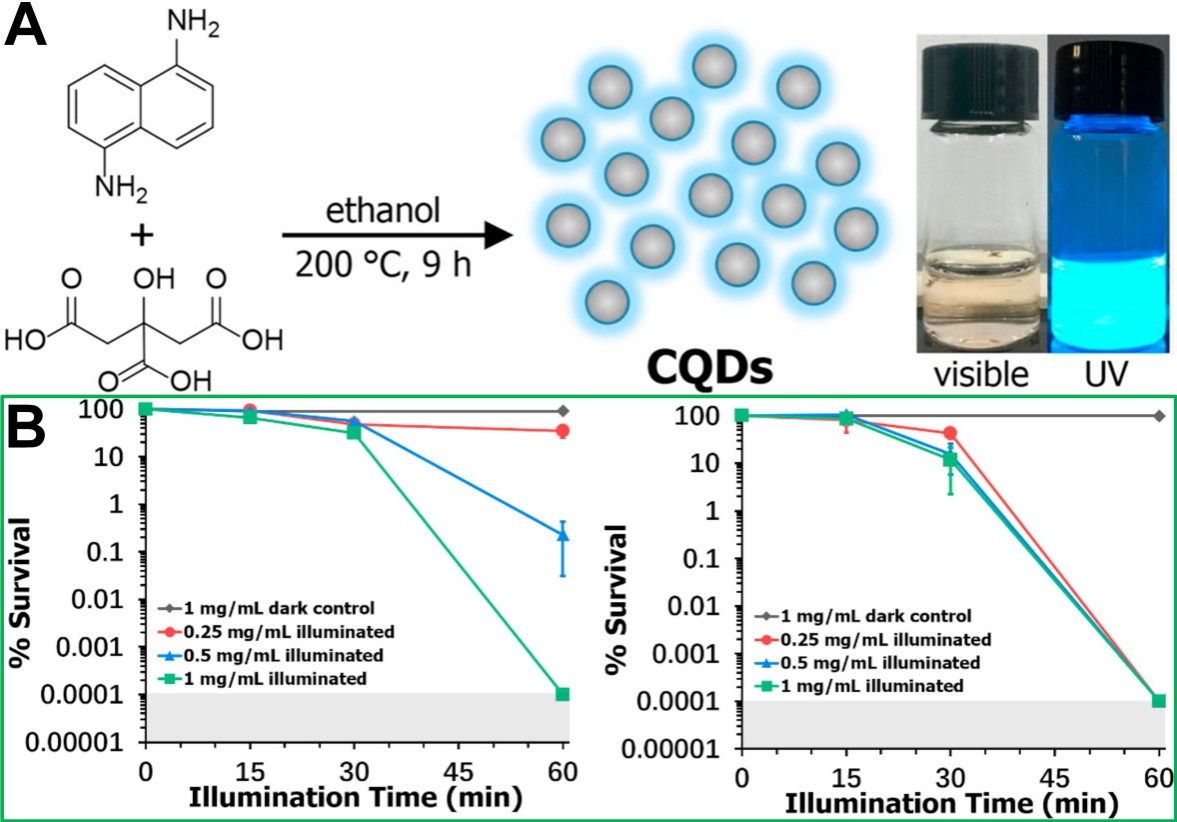
(**A**) Synthesis roadmap of carbon quantum dots (CQDs) and their appearance as an aqueous suspension under visible and UV light illumination. (**B**) Concentration and illumination time dependent photodynamic inactivation studies employing CQDs against* S. aureus* ATCC-6538 and *E. coli* 8099 137. Copyright 2020, Elsevier.

Quantum dots (QDs) have garnered increasing attention as another kind of promising photosensitizers due to their ease-of-synthesis, favourable dispersibility in water, and widely controllable optical properties. Currently, QDs mainly include metal-based (Cd, Pb, and In) semiconductor QDs, carbon QDs and graphene QDs. However, compared with metal-based semiconductor QDs, carbon QDs and graphene QDs are safer, more sustainable, and more biocompatible for potential biomedical applications 130-132. Most GQDs exhibit photothermal performance 133. Recently, it has been reported that GQDs have photodynamic characteristics rather than photothermal effects 134. Carbon QDs (CQDs) can reportedly generate ROS for PDT applications *via* light absorption 136. For example, Wei *et al.* 137 synthesized CQDs from citric acid and 1,5-diaminonaphthalene in ethanol using a one-pot solvothermal method (**Figure 10A**), and the *in vitro* antibacterial photodynamic inactivation of both Gram-positive and Gram-negative bacteria are shown in **Figure 10B.**

## Single-mode phototherapy

### Photodynamic treatment

Photodynamic treatment is a photochemistry-based technology that relies on the generation of singlet oxygen (^1^O_2_) and ROS under light irradiation in the presence of photosensitizers and oxygen 138, and has been reported to be effective in killing both Gram-negative and Gram-positive bacteria 139-141. It is generally believed that photosensitizers can undergo type I (electron transfer) and/or type II (energy transfer) processes to produce highly reactive ROS under light irradiation 142. Type I processes produce radical and radical anion species (*e.g.,* O_2_^-^, and ·HO), while type II processes generate ^1^O_2_ 143. However, antioxidant molecules, for example, glutathione, are present in the complex abscesses site, which could protect bacteria from the toxicity of photodynamic treatment. In addition, the hypoxic microenvironment of an infection site limits the efficacy of oxygen-dependent photodynamic treatment to kill bacteria 144. Moreover, most existing photosensitizers (*e.g.,* PpIX) often aggregate in aqueous solution because of their large conjugated molecular structures. This aggregation usually results in a lack or low levels of ROS generation due to aggregation-caused quenching, which severely hampers the application of photosensitizers in photodynamic treatment 25. Herein, Yoon *et al.* found that boronic acid-functionalized phthalocyanine (PcN4-BA) displays an uncommon phenomenon, an aggregation-enhanced photodynamic effect. The combination of the ability to form uniform nanostructured self-assemblies in water, highly efficient ROS generation and boronic acid-induced targeting cause PcN4-BA to exhibit excellent performances in antimicrobial photodynamic treatment 145.

### Photothermal treatment

The mechanism of photothermal antibacterial treatment occurs by producing local high heat through the phototherapy agent, which can cause the irreversible ablation of bacteria by the denaturation of their proteins/enzymes, inhibiting their essential intracellular reactions 146, 147. Therefore, photothermal nanomaterials have been proposed as a promising solution for the targeted treatment of pathogenic micro-organisms as they are controllable and can be localized to the immediate area surrounding the nanomaterial 148, 149. Currently, NIR laser-triggered photothermal treatment based on various nanoagents has become one of the most effective antibacterial strategies due to the high spatial resolution and tissue penetration depth of NIR lasers 104, 150. Additionally, NIR lasers can be focused on a target area to promote blood circulation and relieve inflammation of tissues. However, there are still some limitations to photothermal treatment alone, whose antibacterial effect is restricted by the following issues: 1) high power density and long-term exposure of NIR lasers can damage healthy tissues 151; 2) it is difficult to kill the heat-resistant bacteria 152; 3) the phototherapy agent cannot rapidly and effectively eradicate bacteria due to its poor diffusivity 153. Photothermal treatment-based synergistic therapy (*e.g.,* a combination with chemotherapy or gas therapy) is a promising strategy for addressing these limitations and integrates the advantages of single modality approaches to shorten antibacterial time and improve antibacterial efficiency 35.

### Advantages and disadvantages of phototherapy

Compared to traditional antibiotic therapy, both photodynamic treatment and photothermal treatment have several advantages in bacterial infections therapy. For example, treatment strategy can be adjusted in a timely manner according to the actual situation since the irradiation site and time can be precisely controlled. Notably, the limited irradiation area can reduce side effects and ensure more locally concentrated energy, which would significantly improve the therapeutic effect. However, despite these advantages, there are still disadvantages. For photodynamic treatment, the high concentrations of glutathione and hypoxic microenvironment at the infection site can limit the efficacy of ROS-dependent photodynamic treatment to kill bacteria. For photothermal treatment, it is difficult to kill the heat-resistant bacteria even when treated at high temperatures such as 45 °C. Moreover, so far, a wide range of the clinical application of single-mode phototherapy is limited due to the poor tissue penetration of light source. Fortunately, the second NIR window (NIR-II, 1000-1700 nm) provides a solution for deep-tissue therapy and diagnosis. Therefore, the development of photosensitizers based on the second NIR window may be the focus of future research.

## Combined phototherapy

To address the intractability of bacteria, single-mode therapy is insufficient for clinical use. Phototherapy combined with other therapeutic methods provides a better alternative for infectious therapy through their respective therapeutic advantages.

### Combination with chemotherapy

At present, antibiotics used as the main chemotherapy drugs are used in the treatment of bacterial infection 154. It is well known that oral and intravenous administrations are the two main forms of antibiotic treatment, which may lead to only a small proportion of the drug being concentrated at the site of the lesion, resulting in less effective treatment 44. In order to maintain the effective treatment concentration of antibiotics at the infected site and ensure the treatment effect, it is often necessary to increase the dosage of drugs or prolong the treatment cycle. However, large dose or long time of drug use would enhance the harmful effects of drugs in the human body and produce severe side effects. The common toxicity and side effects of antibiotics are shown in **Table 5**, mainly involving the gastrointestinal tract, liver, kidney and other organs. To overcome these difficulties, chemotherapy in combination with phototherapy has been significantly emphasized in many recent studies. Nanosystems offer a clue to solving the drawbacks of chemotherapy while introducing a more advanced therapeutic effect 155-157.

**Figure 11:**
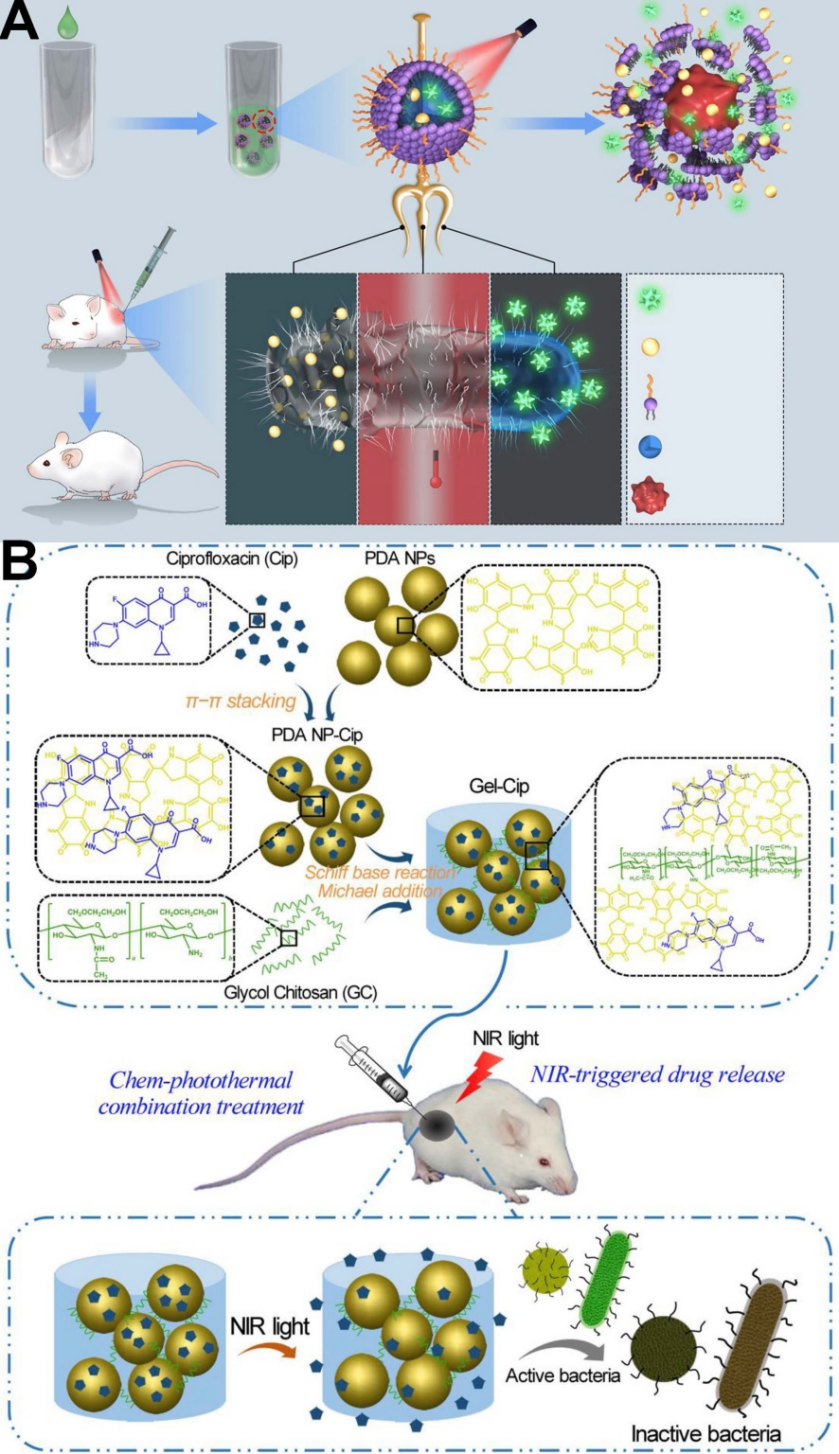
(**A**)Schematics of NIR-activated thermo-responsive-inspired drug-delivery nanotransporter (TRIDENT) for killing antibiotic-resistant bacteria 20. Copyright 2019, Nature Publishing Group. (**B**) Synthetic route of PDA NP-Cip/GC hydrogel and NIR light irradiation-triggered Cip release from PDA NP-Cip/GC hydrogel for bacterial inactivation, Cip: Ciprofloxacin; PDA: Polydopamine; GC: glycol chitosan 160. Copyright 2019, Elsevier.

Nanoparticles such as AuNPs, graphene, and other polymer nanoparticles capable of photothermal conversion have been designed to deliver antibiotics to the infected sites for achieving chem-photothermal therapy under NIR irradiation 118, 158,159. These synergetic strategies can not only fully exploit the advantages of enhancing the antibacterial effect, but also reduce the side effects of antibiotics. For example, Liang *et al.* 20 developed a smart biocompatible thermo-responsive-inspired drug-delivery nanotransporter (TRIDENT) for the synergistic eradication of MDR bacteria through combined chemo-photothermal therapy. As shown in **Figure 11A**, under NIR irradiation, the prepared imipenem (IMP)-loaded IR780 TRIDENT (IMP/IR780@TRN) could increase the local temperature to kill the bacteria under NIR irradiation. IMP/IR780@TRN can melted at temperatures above 43 °C, which causes IMP to be released from the NPs and damage the MDR bacteria. Even low doses of IMP-encapsulated TRIDENT could eradicate clinical MRSA according to *in vitro* and *in vivo* evidence. Therefore, this combination therapy strategy can reduce the use of antibiotics to prevent MDR while ensuring efficacy.

In another report, Wu *et al.* 160 designed a chemo-photothermal combination therapy system (PDA NP-Cip/GC hydrogel, abbreviated as Gel-Cip). As **Figure 11B** shows, the phototherapy agent polydopamine (PDA) NPs were firstly used to load the antibiotic ciprofloxacin (Cip) *via* π-π stacking and/or hydrogen bonding interactions, and then the Cip-loaded PDA NPs was mixed with glycol chitosan (GC) to form an injectable hydrogel through Schiff base reaction and/or Michael addition. Both *in vitro* and *in vivo* evidence confirmed that this combination treatment of Gel-Cip and NIR light irradiation could eradicate both Gram-positive and Gram-negative bacteria.

### Combination with chemodynamic therapy

**Figure 12:**
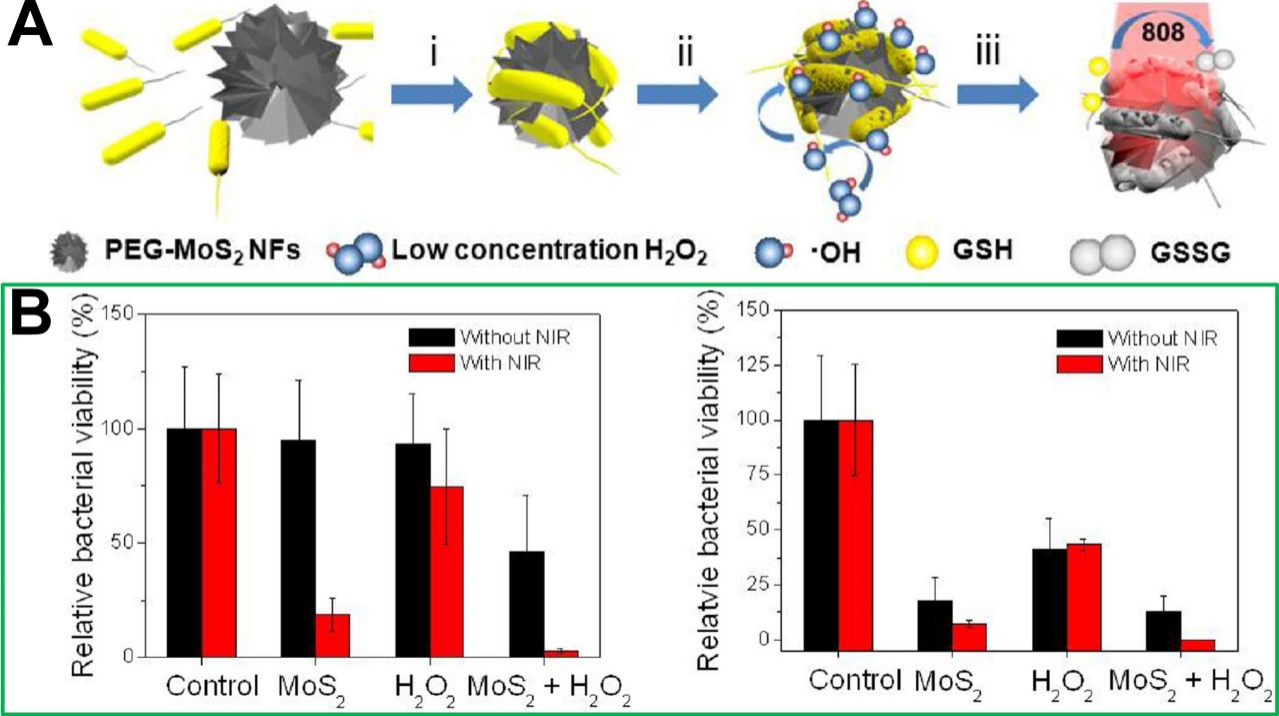
(**A**) Synthetic route of polyethylene glycol functionalized molybdenum disulfide nanoflowers (PEG-MoS_2_ NFs). (**B**) Relative bacteria viabilities of *E. coli* and *B. subtilis* after incubation with different conditions without or with NIR irradiation 101. Copyright 2016, ACS Publications.

Although chemo-photothermic combined therapy strategies can significantly improve the effect of photothermal treatment and reduce the dosage of antibiotics, the use of antibiotics has the risk of producing drug-resistant bacteria. To avoid the occurrence of drug-resistant bacteria, and improve the effect of photothermal treatment at the same time, non-antibiotic combined photothermal treatment strategies have been developed. To our best knowledge, the less reactive H_2_O_2_ can be converted into a highly reactive hydroxyl radical (·OH) through the iron-mediated Fenton chemistry, which will cause the oxidative stress and alteration of biomolecules (*e.g.,* proteins and DNA) 161, 162. It has been discovered that there is a significant overproduction of H_2_O_2_ in the microenvironment of bacterial infection site 163. Therefore, the biofilm formation of bacteria could be prevented by applying ferromagnetic NPs to produce ·OH 164. In addition, nanoenzymes are nanomaterials with peroxidase- and oxidase-like properties, and also have been explored as powerful tools to kill bacteria due to their capability to catalyze the formation of ROS 164-167. For instance, noble metal NPs, such as Au, Pd, and Pt, have attracted extensive attention in the antibacterial field 168-171. Though this is an inspiring protocol with promising antibacterial outcomes, the existing metal-carrying nanomaterials often have low reaction rates due to the insufficient catalyst ion amount, which results in slow ·OH generation and unsatisfactory chemodynamic therapy outcome 172-175. Therefore, it is necessary to design multimode antibacterial nanomaterials with chemodynamic therapeutic effects. For example, Zhao *et al.* 101 combined catalysis with the NIR photothermal effect and developed a biocompatible antibacterial system based on polyethylene glycol functionalized molybdenum disulfide nanoflowers (PEG-MoS_2_ NFs). *In vitro* experiments confirmed that this combination therapy system could provide a faster and more effective kill outcome for Gram-negative ampicillin resistant *Escherichia coli* and Gram-positive endospore-forming *Bacillus subtilis* than chemodynamic treatment or photothermal treatment alone (**Figure 12**).

In addition, to address the impact of low oxygen content in the infection microenvironment during antimicrobial photodynamic treatment, Liu *et al.* 21 utilized manganese dioxide (MnO_2_), since it could effectively catalyze H_2_O_2_ conversion into H_2_O and O_2_ 176, 177 to prepare a photosensitizer (Ce6)-carrying MnO_2_ nanometre system (Ce6@MnO_2_-PEG NPs). Owing to the ability of MnO_2_ to convert H_2_O_2_ into O_2_, Ce6@MnO_2_-PEG NPs could greatly enhance the photodynamic treatment-induced antibacterial efficacy within the oxygen-deficient/H_2_O_2_-enriched environment compared to free Ce6.

### Combination with gas therapy

**Figure 13:**
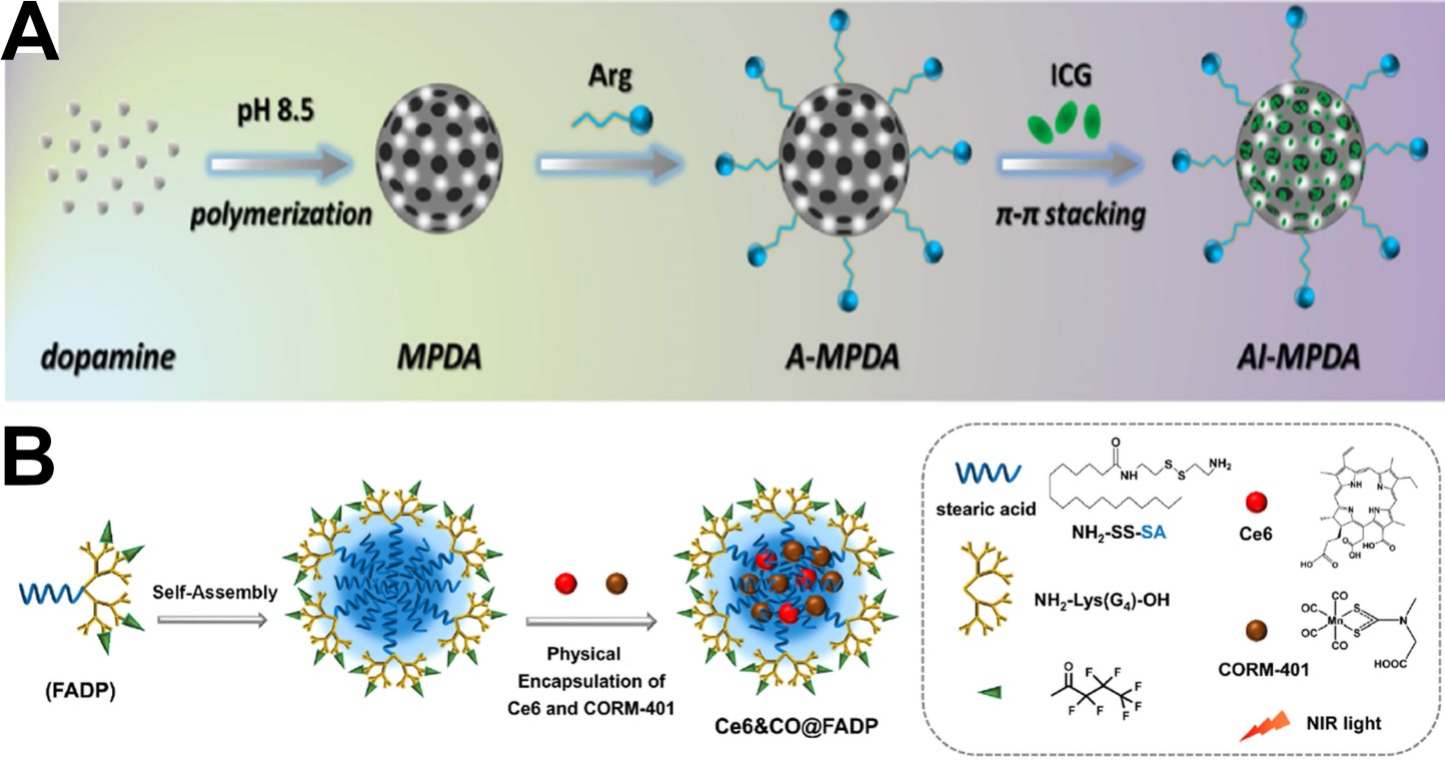
(**A**) Synthetic route of ICG-loaded L-arginine conjugate mesoporous polydopamine nanoparticles (AI-MPDA) 28. Copyright 2018, Wiley-VCH. (**B**) The schematic illustration for the preparation of Ce6&CO@FADP, FADP: fluorinated amphiphilic dendritic peptide 191. Copyright 2020, ACS publication.

Recently, due to the therapeutic efficiency, mild side effect, and rare chance to induce bacterial MDR, gas therapy has attracted enormous attention in antibacterial applications 178. Among the gases used, NO is the one that has been most extensively explored due to its multi-antibacterial mechanisms 179, 180, including inhibiting bacterial growth by reacting with bacterial DNA and blocking the repair of damaged DNA, and reacting with ROS to generate reactive peroxynitrite (ONOO-) molecules with enhanced bactericidal activity. However, a high concentration of gas can inevitably damage *in vivo* normal tissues 181, 182. Therefore, photothermal treatment combined with gas therapy formed another kind of strategy for antimicrobial therapy. For example, Gao* et al.* 183 reported a new near-infrared 808 nm laser-mediated NO-releasing nanovehicle (MoS_2_-BNN6), that could not only exhibit photothermal treatment efficacy but also precisely control NO release to generate oxidative/nitrosative stress under NIR irradiation (λ=808 nm).* In vivo* results showed that MoS_2_-BNN6 with enhanced photothermal treatment/NO synergetic antibacterial function achieves >97.2% inactivation of bacteria within 10 min of NIR irradiation, and could also effectively repair wounds through the formation of collagen fibres and elimination of inflammation during tissue reconstruction. Similarly, Cai *et al.* 28 presented an all-in-one phototherapeutic nanoplatform AI-MPDA, which includes L-arginine (L-Arg), ICG and mesoporous polydopamine (MPDA) (**Figure 13A**). AI-MPDA not only generated heat but also produced ROS and catalyzed L-Arg to release NO under NIR irradiation, furthermore, the released NO significantly enhanced the therapeutic effects of photodynamic treatment and low-temperature photothermal treatment (≤45 °C), contributing to the elimination of bacterial biofilm. Different from NO gas, hydrogen (H_2_) can selectively scavenge intracellular ROS without inducing any toxic effect on normal cells even at high concentrations 184, 185, which has been recognized as a green, safe and excellent antioxidant combined photothermal treatment in antibacterial and wound-healing therapies. For example, Xue *et al.* 186 reported a NIR-mediated hydrogen release photothermal agent, PdH nanohydride, which combined both merits of bioactive hydrogen and the photothermal effect of Pd, exhibiting excellent antibacterial activities *in vitro* and *in vivo* due to its synergistic hydrogen-photothermal antibacterial process occurring through oxidative stress and membrane damage pathway.

Additionally, carbon monoxide (CO) has been considered a potent antibacterial agent against diverse microorganisms 187 *via* the p38-mediated surface expression of toll-like receptor 4 (TLR-4), activation of host immune responses 188, and inhibition of cellular respiration 189. Furthermore, it even exhibits a high eradication effect on bacterial biofilms 190. Therefore, CO therapy has been also combined with phototherapy for antibacterial treatment. Cai *et al.* developed a photodynamic treatment-driven CO-controlled delivery system (Ce6&CO@FADP) by the chemical conjugation of Ce6 and physical encapsulation of CORM-401 into peptide dendrimer nanogels 191. The schematic illustration of the preparation of Ce6&CO@FADP can be found in** Figure 13B.** The *In vitro* antibacterial effect shows that the survival rate of the Ce6&CO@FADP-treated *E. coli* was only 0.5%, which is significantly lower than that achieved by other treatment groups. Therefore, the combination of CO and photodynamic treatment has a good prospect in bacterial infection treatment.

### Multiple combination therapy

**Figure 14:**
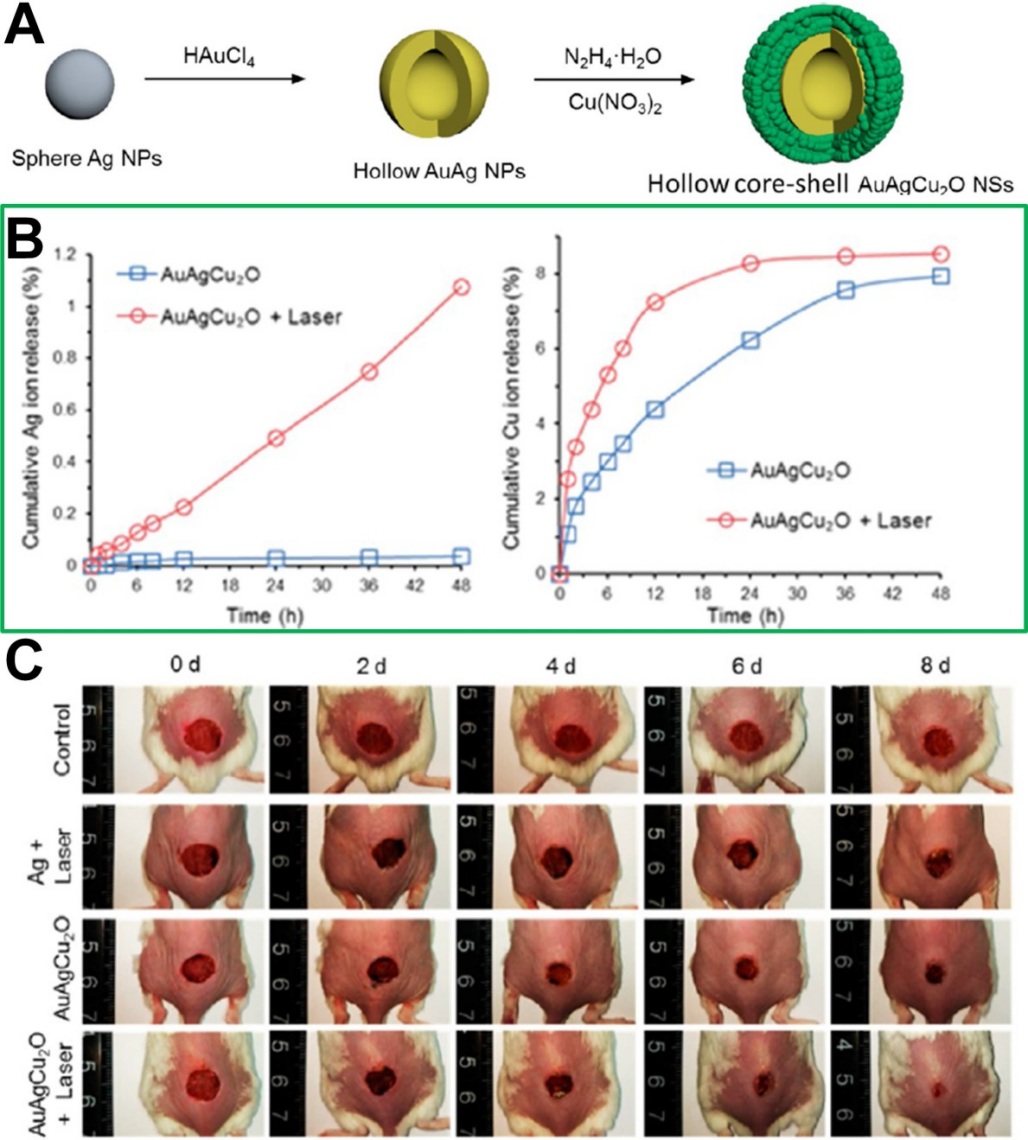
(**A**) Synthetic route of AuAgCu_2_O NSs (a hollow gold-silver (AuAg) core and Cu_2_O shell). (**B**) Cumulative release amounts of Ag and Cu ions from AuAgCu_2_O NS hydrogel suspension. (**C**) Representative macroscopic appearance of MRSA-infected full-thickness dorsal cutaneous incisions on BALB/c mice disposed by diverse treatments^192^. Copyright 2020, ACS publications.

Because multi-channel antibacterial therapy has good antibacterial effects with the continuous development of nanomedicine, multiple combination therapy strategies have been developed and used in bacterial infection treatment in recent years. The heat induced by photothermal treatment can not only increase the ambient temperature of tissue to achieve antimicrobial efficacy but also increase blood flow and oxygen supply to enhance the ^1^O_2_ generation of photodynamic treatment, and so the multiple treatment strategy combined with photodynamic and photothermal therapy achieves a better antibacterial effect than that achieved by the individual therapies alone. For example, Zhang *et al.* 135 utilized the intrinsic antibacterial action of chitosan oligosaccharide (COS) and the photodynamic and photothermal properties of graphene quantum dots (GQDs) to prepare COS functionalized GQDs (GQDs-COS) that exhibit multiple synergetic antibacterial activities, and could interact with negatively charged bacterial surfaces *via* electrostatic attraction. Additionally, under 450 nm light irradiation, GQDs-COS could simultaneously kill Gram-positive and Gram-negative bacteria *via* three types of antibacterial activity (photodynamic, photothermal and chemical therapy). In addition, as wound repair is very important in local bacterial infection treatment, Zhou *et al.* 192 reported hybrid hollow core-shell heterostructured AuAgCu_2_O NSs, including a hollow gold-silver (AuAg) core and Cu_2_O shell. The AuAgCu_2_O NSs have been used as a photothermal therapeutic agent for cutaneous chronic wound and nonhealing keratitis with drug resistant bacterial infection. As shown in **Figure 14**, under NIR irradiation, the silver ion could be released from the hollow AuAg core and cooperate with the photothermal treatment of Au to eradicate MDR bacteria, including extended-spectrum β-lactamase *Escherichia coli* and MRSA. In addition, wound-healing effects could be boosted because of the rapid endothelial cell angiogenesis and fibroblast cell migration achieved by the copper ion released from the Cu_2_O shell.

## Conclusions and perspectives

Intensive efforts have been devoted to the development of phototherapy-based antibacterial nanomaterials to deal with bacterial infections. In this review, we first summarized recent commonly used phototherapy agents, including organic phototherapy agents (*e.g.,* small molecule photosensitizers and loaded-photosensitizer nanoparticles) and inorganic phototherapy agents (metal nanomaterial, upconversion nanoparticles and quantum dots). Then the advantages and disadvantages of single mode photothermal treatment were analyzed by introducing their specific situations. Moreover, to address the limitations of photothermal therapy in bacterial infection treatment, many combination therapy strategies have emerged, such as combination with chemotherapy, combination with chemodynamic therapy, combination with gas therapy and multiple combination therapy. In particular, combination with chemodynamic therapy and gas therapy can solve the problem of MDR resistance very well due to the lack of antibiotics used in the combination therapy system.

In the future, only very biocompatible antibacterial nanomaterials are promising for potential clinical application. Therefore, although these combined strategies have shown promising results in the treatment of infectious diseases, many important fundamental issues of nanomaterials must be studied in depth and fully elaborated, including the synergistic action mechanisms, accumulation of lesions, metabolic pathways and toxicities toward vital organs. More importantly, before designing nanomedicine, it is necessary to consider how to use them in patients (*e.g.,* intravenous injection, oral, subcutaneous injection or spray). Additionally, for deep tissue infection, phototherapy cannot be used unless the site of infection has been identified, which severely limits its clinical use in antibacterial infections treatment. Moreover, a consensus has been reached among researchers worldwide that the patient survival rate can be greatly increased if the disease is effectively diagnosed and treated at the early stage 193, 194. Based on this investigation, Wang *et al.* reported a magnetic nanosystem combined with photodynamic treatment, which could identify bacterial species for early sepsis diagnosis and extracorporeal photodynamic blood disinfection 29. Therefore, designing a phototherapy-based nanoplatform with integrated diagnosis and treatment abilities is a very promising strategy to address bacterial infections and should have good imaging capabilities (*e.g.,* magnetic resonance imaging, fluorescence imaging, ultrasound, etc.) at the infected site to accurately guide light to the site, to prevent toxicity to normal tissues during the phototherapy process.

## Figures and Tables

**Table 1:** Small molecule photosensitizers-loaded nanoparticles for antibacterial activities

Type	Photosensitizer	Loading method	Phototherapy type	Target microbe	Ref.
Vesicle	Ce6	Conjugate	Photodynamic treatment	*S. aureus*	19
	IR-780	Encapsulation	Photodynamic treatment and photothermal treatment	MRSA	20
Polymer nanoparticles	Ce6	Encapsulation	Photodynamic treatment	MRSA	21
	ICG	Encapsulation	Photodynamic treatment	*P. aeruginosa*	22
	PpIX	Encapsulation	Photodynamic treatment	*S. aureus*	23
Silica nanoparticles	Ce6	Conjugate	Photodynamic treatment	MRSA	24-26
MnO_2_ nanoparticles	Ce6	Encapsulation	Photodynamic treatment	*S. aureus*	27
Mesoporous Polydopamine nanoparticles	ICG	Encapsulation	Photodynamic treatment and photothermal treatment	*S. aureus*	28
F_3_O_4_ nanoparticles	Ce6	Conjugate	Photodynamic treatment	*S. aureus* *E. coli*	29
Nanoscale metal-organic frameworks	Squaraine	Encapsulation	Photodynamic treatment	*S. aureus*	30
Carboxymethyl chitosan nanoparticles	MB	Encapsulation	Photodynamic treatment	*E. coli, S. aureus,* MRSA	31
Nanofiber	Cypate	Encapsulation	Photothermal treatment	MRSA	44
UCNPs	SiPc	Encapsulation	Photodynamic treatment	*E. coli, S. aureus*	32
	MB	Encapsulation	Photodynamic treatment	*S. aureus, E. coli*	33
	MB	Encapsulation	Photodynamic treatment	*S. aureus, E. coli*	34
	ZnPc	Encapsulation	Photodynamic treatment	*S. aureus*	35
	CPZ	Conjugate	Photodynamic treatment	MRSA	15
	PSeV	Encapsulation	Photodynamic treatment and photothermal treatment	MRSA	36
	Curcumin	Conjugate	Photodynamic treatment	MRSA	37

**Table 2:** Comparison of photothermal carbon nanomaterials for antibacterial activities

Material	Shape	Irradiation (λ)	Target microbe	Ref.
GCS-CG	Sheet	808 nm	*E. coli* and* S. aureus*	54
Ag@G-SAS	Sheet	808 nm	*E. coli* and *S. aureus*	56
FGO-FA	Sheet	808 nm	*E. coli* and* S. aureus*	57
AA@GS@HA-MNPs	Sheet	808 nm	*E. coli* and *S. aureus*	58
GO-IO-CS	Sheet	808 nm	*E. coli* and *S. aureus*	59
Van-rGO	Sheet	808 nm	*E. coli*	60
MRGOGA	Sheet	808 nm	*E. coli* and* S. aureus*	61
GAS-Ab-MWNT	Tube	800 nm	*Planktonic* and *GAS*	62
IgG-MWCNT	Tube	808 nm	MRSA	63

**Table 3:** Comparison of photothermal metal nanomaterials for antibacterial activities

Material	Shape	Irradiation (λ)	Target microbe	Ref.
Au	Sphere	525 nm	*S. aureus*	70
	Popcorn	670 nm	*S. typhimurium*	71
	Oval	670 nm	*S. typhimurium*	72
	Star	808 nm	*S. aureus*	73
	Cluster	808 nm	*E. coli*	74
	Rod	810 nm	*E. coli, E. faecalis, S. aureus, S. mutans, S. oralis, S. salivarius, S. sobrinus.*	75
AuPd	Sheet	808 nm	*S. aureus* and* E. coli*	76
Au@SiO_2_	-----	810 nm	*E. faecalis*	77
AuNSs	Star	808 nm	*MRSA*	78
AuNRs-PVCL gel	Rod	785 nm	*AMR E. coli, AMR A. baumannii, AMR E. faecalis*	79
Au@Ag NPs	Sphere	800 nm	*S. aureus*	80
Au-Ag-Au NRs	Rod	785 nm	*E. coli*	81
Au@PtAg NRs	Rod	808 nm	*E. coli* and* S. aureus*	82
PEG-Au@Ag NPs	Triangle	808 nm	*E. coli*	83
AuNP-N-C	Sphere	808 nm	*MRSA*	84
Apt@AuNRs	Sphere and rod	808 nm	*MRSA*	85
AuNR@HSK	Sphere	785 nm	*E. coli*	86
AuNR@PAA-Van	Rod	808 nm	*E. coli*	87
Tetra@AuNR@TiO_2_	Tubes	830 nm	*S. mutans*	88
Fe_3_O_4_-Au	Eggs	808 nm	*A. baumannii, E. coli, S. pyogenes, S. saprophyticus, E. faecalis and E. faecium*	89
	Necklace	785 nm	*E. coli* and* S. typhimurium*	90
Fe_3_O_4_-alumina	Sphere	808 nm	*A. baumannii, E. coli, E. faecalis* and *S. pyogenes*	91
Fe_5_C_2_	Spheroid	808 nm	*S. aureus* and* E. coli*	92
In_2_Se_3_	Sheets	808 nm	*S. aureus* and* E. coli*	93
CuS	Plate-like	980 nm	*S. aureus* and* E. coli*	94
CuS-PVP	hexagonal nanoplates	808 nm	*S. aureus*	95
BSA-CuS	Platelike or sphere	980 nm;808 nm	*S. aureus, E. coli, A. baumannii, S. haemolyticus, MRSA*	96-98
Ce6-labeled BSA-CuS	Sphere	1064 nm	*S. aureus* and* E. coli*	99
LuVO4:Nd3+/Yb3+/Er3+@SiO_2_@Cu_2_S	Olivelike	808 nm	*S. aureus* and* E. coli*	100
PEG-MoS_2_	Flower-like	808 nm	*Ampr E. coli* and *B. subtilis*	101
CS-Fe_3_O_4_-MoS_2_	Sheet	808 nm	*S. aureus* and *E. coli*	102
MoS_2_-BNN6	Sheet	808 nm	*S. aureus, Ampr E. coli, E. faecalis*	103
MoS_2_-penicillin	Sheet	808 nm	*S. aureus* and* E. coli*	104
MoO_3_-x/Ag	Sheet	808 nm	*S. aureus* and* E. coli*	105
Cs_0.33_WO_3_ on CA-PVP decorated microchannels	Sphere	808 nm	*S. aureus* and* E. coli*	106
GTA-PEG-W_18_O_49_	Sheet	808 nm	*E. coli*	107
Van-LaB_6_@SiO_2_/Fe_3_O_4_	Sphere	808 nm	*S. aureus* and* E. coli*	108
Ag	Triangular	808 nm	*S. aureus* and* E. coli*	69

**Table 4:** Comparison of photothermal composite nanomaterials for antibacterial activities

Material	Irradiation (λ)	Target microbe	Ref.
Ag+-GCS-PDA@AuNRs	808 nm	*E. coli* and* MRSA*	113
Dap-GCS-PDA@AuNRs	808 nm	*MRSA*	114
AuNC@Dap/PDA-aSpa	808 nm	*S. aureus*	115
PDA@Au-Hap NPs	808 nm	*E. coli* and *S. aureus*	116
PATA-C_4_@CuS	980 nm	*S. aureus, B. amyloliquefaciens, P. aeruginosa* and* E. coli*	117
Au@Rubpy/GO	785 nm	*E. coli* and* S. aureus*	110
GO-IONP-Ag	808 nm	*S. aureus*	118
Au-QCMC-GO/nanocellulose paper	808 nm	*E. coli*,* P. aeruginosa* and *B. subtilis*	112
GO-Tob@CuS	980 nm	*E. coli* and* P. aeruginosa*	119
Fe_3_O_4_@GO-QCS	808 nm	*E. coli* and* S. aureus*	120
rGO/AuNS	808 nm	*E. coli* and* S. aureus*	111
rGO/MSN/Ag	808 nm	*E. coli*,* P. putida* and* Rhodococcus*	121
Ag@rGO-Fe_3_O_4_-PEI	785 nm	*E. coli*	122
rGO-Fe_3_O_4_-Au-Ag-Au	785 nm	*E. coli*	123
Ag/ZnO/rGO	808 nm	*E. coli*	124
Fe_3_O_4_-CNT-PNIPAM	808 nm	*E. coli* and *S. aureus*	125
BP/Au	808 nm	*E. faecalis*	129

**Table 5:** The common toxic and side effects of each type of antibiotics

Type	Adverse events
Penicillins	Pain and allergic reactions
Cephalosporins	Allergic reactions
Macrolides	Gastrointestinal side effects, temporary auditory impairment and cardiotoxicity
Tetracyclines	Gastrointestinal side effects and nephrotoxicity
Polymyxins	Nephrotoxicity, allergic reactions and neurotoxicity

## References

[1] Peleg AY, Hooper DC (2010). Hospital-acquired infections due to gram-negative bacteria. N Engl J Med.

[2] Angus DC, Poll van der T (2013). Severe sepsis and septic shock. N Engl J Med.

[3] Mintzer MA, Dane EL, O'Toole GA, Grinstaff MW (2012). Exploiting dendrimer multivalency to combat emerging and re-emerging infectious diseases. Mol Pharm.

[4] Ning X, Lee S, Wang Z, Kim D, Stubblefield B, Gilbert E (2011). Maltodextrin-based imaging probes detect bacteria *in vivo* with high sensitivity and specificity. Nat Mater.

[5] Heemskerk AD, Bang ND, Mai NTH, Chau TTH, Phu NH, Loc PP (2016). Intensified antituberculosis therapy in adults with tuberculous meningitis. N Engl J Med.

[6] Levy SB, Marshall B, Antibacterial resistance worldwide (2004). causes, challenges and responses. Nat Med.

[7] Holmes AH, Moore LSP, Sundsfjord A, Steinbakk M, Regmi S, Karkey A (2016). Understanding the mechanisms and drivers of antimicrobial resistance. Lancet.

[8] Gelband H, Laxminarayan R (2015). Tackling antimicrobial resistance at global and local scales. Trends Microbiol.

[9] Baym M, Lieberman TD, Kelsic ED, Chait R, Gross R, Yelin I (2016). Spatiotemporal microbial evolution on antibiotic landscapes. Science.

[10] Marston HD, Dixon DM, Knisely JM, Palmore TN, Fauci AS (2016). Antimicrobial Resistance. JAMA.

[11] Organization WH Antibiotic resistance: multi-country public awareness survey. 2015.

[12] Xie Z, Fan T, An J, Choi W, Duo Y, Ge Y (2020). Emerging combination strategies with phototherapy in cancer nanomedicine. Chem Soc Rev.

[13] Maisels MJ, McDonagh AF (2008). Phototherapy for neonatal jaundice. N Engl J Med.

[14] Li X, Lovell JF, Yoon J, Chen X (2020). Clinical development and potential of photothermal and photodynamic therapies for cancer. Nat Rev Clin Oncol.

[15] Zhang Y, Huang P, Wang D, Chen J, Liu W, Hu P (2018). Near-infrared-triggered antibacterial and antifungal photodynamic therapy based on lanthanide-doped upconversion nanoparticles. Nanoscale.

[16] Tao W, Kong N, Ji X, Zhang Y, Sharma A, Ouyang J (2019). Emerging two-dimensional monoelemental materials (Xenes) for biomedical applications. Chem Soc Rev.

[17] Qiu M, Ren WX, Jeong T, Won M, Park GY, Sang DK (2018). Omnipotent phosphorene: a next-generation, two-dimensional nanoplatform for multidisciplinary biomedical applications. Chem Soc Rev.

[18] Li L, Ma H, Qi G, Zhang D, Yu F, Hu Z (2016). Pathological-condition-driven construction of supramolecular nanoassemblies for bacterial infection detection. Adv Mater.

[19] Zhang R, Li Y, Zhou M, Wang C, Feng P, Miao W (2019). Photodynamic chitosan nano-assembly as a potent alternative candidate for combating antibiotic-resistant bacteria. ACS Appl Mater Interfaces.

[20] Qing G, Zhao X, Gong N, Chen J, Li X, Gan Y (2019). Thermo-responsive triple-function nanotransporter for efficient chemo-photothermal therapy of multidrug-resistant bacterial infection. Nat Commun.

[21] Wijesiri N, Ozkaya-Ahmadov T, Wang P, Zhang J, Tang H, YuOrcid X (2017). Photodynamic inactivation of multidrug-resistant *Staphylococcus aureus* using hybrid photosensitizers based on amphiphilic blockcopolymer-functionalized gold nanoparticles. ACS Omega.

[22] Zhao Y, Yu C, Yu Y, Wei X, Duan X, Dai X (2019). Bioinspired heteromultivalent ligand-decorated nanotherapeutic for enhanced photothermal and photodynamic therapy of antibiotic-resistant bacterial pneumonia. ACS Appl Mater Interfaces.

[23] Liu Y, Mei van der HC, Zhao B, Zhai Y, Cheng T, Li Y, Zhang Z (2017). Eradication of multidrug-resistant staphylococcal infections by light-activatable micellar nanocarriers in a murine model. Adv Funct Mater.

[24] Lin J, Li J, Gopal A, Munshi T, Chu Y, Wang J (2019). Synthesis of photo-excited Chlorin e6 conjugated silica nanoparticles for enhanced anti-bacterial efficiency to overcome methicillin-resistant *Staphylococcus aureus*. Chem Commun.

[25] Zhao Z, Yan R, Wang J, Wu H, Wang Y, Chen A (2017). A bacteria-activated photodynamic nanosystem based on polyelectrolyte-coated silica nanoparticles. J Mater Chem B.

[26] Wang C, Chen P, Qiao Y, Kang Y, Yan C, Yu Z (2020). pH responsive superporogen combined with PDT based on poly Ce6 ionic liquid grafted on SiO_2_ for combating MRSA biofilm infection. Theranostics.

[27] Wang C, Xiao Y, Zhu W, Chu J, Xu J, Zhao H (2020). Photosensitizer-modified MnO_2_ nanoparticles to enhance photodynamic treatment of abscesses and boost immune protection for treated mice. Small.

[28] Yuan Z, Lin C, He Y, Tao B, Chen M, Zhang J (2020). Near-infrared light-triggered nitric-oxide-enhanced photodynamic therapy and low-temperature photothermal therapy for biofilm elimination. ACS Nano.

[29] Wang J, Wu H, Yang Y, Yan R, Zhao Y, Wang Y (2018). Bacterial species-identifiable magnetic nanosystems for early sepsis diagnosis and extracorporeal photodynamic blood disinfection. Nanoscale.

[30] Bagchi D, Bhattacharya A, Dutta T, Nag S, Wulferding D, Lemmens P (2019). Nano MOF entrapping hydrophobic photosensitizer for dual-stimuli-responsive unprecedented therapeutic action against drug-resistant bacteria. ACS Appl Bio Mater.

[31] Sun L, Jiang W, Zhang H, Guo Y, Chen W, Jin Y (2019). Photosensitizer-loaded multifunctional chitosan nanoparticles for simultaneous *in situ* imaging, highly efficient bacterial biofilm eradication, and tumor ablation. ACS Appl Mater Interfaces.

[32] Grüner MC, Arai MS, Carreira M, Inada N, Camargo de ASS (2018). Functionalizing the mesoporous silica shell of upconversion nanoparticles to enhance bacterial targeting and killing via photosensitizer-induced antimicrobial photodynamic therapy. ACS Appl Bio Mater.

[33] Sun J, Zhang P, Fan Y, Zhao J, Niu S, Song L (2019). Near-infrared triggered antibacterial nanocomposite membrane containing upconversion nanoparticles. Mater Sci Eng C.

[34] Yin M, Li Z, Ju E, Wang Z, Dong K, Ren J (2014). Multifunctional upconverting nanoparticles for Near-infrared triggered and synergistic antibacterial resistance therapy. Chem Commun.

[35] Li S, Cui S, Yin D, Zhu Q, Ma Y, Qian Z (2017). Dual antibacterial activities of a chitosan-modified upconversion photodynamic therapy system against drug-resistant bacteria in deep tissue. Nanoscale.

[36] Zhou K, Qiu X, Xu L, Li G, Rao B, Guo B (2020). Poly(selenoviologen)-assembled upconversion nanoparticles for low-power single-NIR light-triggered synergistic photodynamic and photothermal antibacterial therapy. ACS Appl Mater Interfaces.

[37] Liu J, Yu M, Zeng G, Cao J, Wang Y, Ding T (2018). Dual antibacterial behavior of a curcumin-upconversion photodynamic nanosystem for efficient eradication of drug-resistant bacteria in a deep joint infection. J Mater Chem B.

[38] Dong X, Chu D, Wang Z (2018). Neutrophil-mediated delivery of nanotherapeutics across blood vessel barrier. Ther Deliv.

[39] Dong X, Chu D, Wang Z (2017). Leukocyte-mediated delivery of nanotherapeutics in inflammatory and tumor sites. Theranostics.

[40] Chu D, Dong X, Zhao Q, Gu J, Wang Z (2017). Photosensitization priming of tumor microenvironments improves delivery of nanotherapeutics via neutrophil infiltration. Adv Mater.

[41] Ding X, Wang A, Tong W, Xu F (2019). Biodegradable antibacterial polymeric nanosystems: a new hope to cope with multidrug-resistant bacteria. Small.

[42] Radovic-Moreno AF, Lu TK, Puscasu VA, Yoon CJ, Langer R, Farokhzad OC (2012). Surface charge-switching polymeric nanoparticles for bacterial cell wall-targeted delivery of antibiotics. ACS Nano.

[43] Zhang CY, Gao J, Wang Z (2018). Bioresponsive nanoparticles targeted to infectious microenvironments for sepsis management. Adv Mater.

[44] Wang J, Chen X, Zhao Y, Yang Y, Wang W, Wu C (2019). pH-Switchable antimicrobial nanofiber networks of hydrogel eradicate biofilm and rescue stalled healing in chronic wounds. ACS Nano.

[45] Naha PC, Liu Y, Hwang G, Huang Y, Gubara S, Jonnakuti V (2019). Dextran-coated iron oxide nanoparticles as biomimetic catalysts for localized and pH-activated biofilm disruption. ACS Nano.

[46] Zhou B, Shi B, Jin D, Liu X (2015). Controlling upconversion nanocrystals for emerging applications. Nat Nanotechnol.

[47] Chen Z, Yuan H, Liang H (2017). Synthesis of multifunctional cationic poly(p-phenylenevinylene) for selectively killing Bacteria and lysosome-specific imaging. ACS Appl Mater Interfaces.

[48] Corbitt TS, Sommer JR, Chemburu S, Ogawa K, Ista LK, Lopez GP (2009). Conjugated polyelectrolyte capsules: light-activated antimicrobial micro “Roach Motels”. ACS Appl Mater Interfaces.

[49] Ding L, Chi EY, Schanze KS, Lopez GP, Whitten DG (2010). Insight into the mechanism of antimicrobial conjugated polyelectrolytes: lipid headgroup charge and membrane fluidity effects. Langmuir.

[50] Sun H, Yin B, Ma H, Yuan H, Fu B, Liu L (2015). Synthesis of a novel quinoline skeleton introduced cationic polyfluorene derivative for multimodal antimicrobial application. ACS Appl Mater Interfaces.

[51] Wang Y, Li S, Liu L, Feng L (2018). Photothermal-responsive conjugated polymer nanoparticles for the rapid and effective killing of bacteria. ACS Appl Bio Mater.

[52] Cheeseman S, Christofferson AJ, Kariuki R, Cozzolino D, Daeneke T, Crawford RJ (2020). Antimicrobial metal nanomaterials: from passive to stimuli-activated applications. Adv Sci.

[53] Xu J, Yao K, Xu Z (2019). Nanomaterials with a photothermal effect for antibacterial activities: an overview. Nanoscale.

[54] Qian W, Yan C, He D, Yu X, Yuan L, Liu M (2018). pH-triggered charge-reversible of glycol chitosan conjugated carboxyl graphene for enhancing photothermal ablation of focal infection. Acta Biomateri.

[55] Zhang Y, Pi Y, Hua Y, Xie J, Wang C, Guo K (2020). Bacteria responsive polyoxometalates nanocluster strategy to regulate biofilm microenvironments for enhanced synergetic antibiofilm activity and wound healing. Theranostics.

[56] Fan X, Yang F, Nie C, Yang Y, Ji H, He C (2018). Mussel-inspired synthesis of NIR-responsive and biocompatible Ag-graphene 2D nanoagents for versatile bacterial disinfections. ACS Appl Mater Interfaces.

[57] Gong P, Wang F, Guo F, Liu J, Wang B, Ge X (2018). Fluorescence turn-off Ag/fluorinated graphene composites with high NIR absorption for effective killing of cancer cells and bacteria. J Mater Chem B.

[58] Ji H, Dong K, Yan Z, Ding C, Chen Z, Ren J (2016). Bacterial hyaluronidase self-triggered prodrug release for chemo-photothermal synergistic treatment of bacterial infection. Small.

[59] Jia X, Ahmad I, Yang R, Wang C (2017). Versatile graphene-based photothermal nanocomposites for effectively capturing and killing bacteria, and for destroying bacterial biofilms. J Mater Chem B.

[60] Yang X, Li Z, Ju E, Ren J, Qu X (2014). Reduced graphene oxide functionalized with a luminescent rare-earth complex for the tracking and photothermal killing of drug-resistant bacteria. Chem -Eur J.

[61] Wu M, Deokar AR, Liao J, Shih P, Ling Y (2013). Graphene-based photothermal agent for rapid and effective killing of bacteria. ACS Nano.

[62] Levi-Polyachenko N, Young C, MacNeill C, Braden A, Argenta L, Reid S (2014). Eradicating group a streptococcus bacteria and biofilms using functionalised multi-wall carbon nanotubes. Int J Hyperthermia.

[63] Yang X, Li Z, Ju E, Ren J, Qu X (2014). Reduced graphene oxide functionalized with a luminescent rare-earth complex for the tracking and photothermal killing of drug-resistant bacteria. Chemistry.

[64] Yu Y, Williams JD, Willets KA (2018). Quantifying photothermal heating at plasmonic nanoparticles by scanning electrochemical microscopy. Faraday Discuss.

[65] Webb JA, Bardhan R (2014). Emerging advances in nanomedicine with engineered gold nanostructures. Nanoscale.

[66] Borzenkov M, Moros M, Tortiglione C, Bertoldi S, Contessi N, Faré S (2018). Fabrication of photothermally active poly(vinyl alcohol) films with gold nanostars for antibacterial applications. Nanotechnol.

[67] Mocan L, Tabaran FA, Mocan T, Pop T, Mosteanu O, Agoston-Coldea L (2017). Laser thermal ablation of multidrug-resistant bacteria using functionalized gold nanoparticles. Int J Nanomed.

[68] Millenbaugh NJ, Baskin JB, DeSilva MN, Elliott WR, Glickman RD (2015). Photothermal killing of Staphylococcus aureus using antibody-targeted gold nanoparticles. Int J Nanomed.

[69] Zhu C, Shen H, Liu H, Lv X, Li Z, Yuan Q (2018). Solution-processable two-dimensional In_2_Se_3_ nanosheets as efficient photothermal agents for elimination of bacteria. Chem -Eur J.

[70] Zharov VP, Mercer KE, Galitovskaya EN, Smeltzer MS (2006). Photothermal nanotherapeutics and nanodiagnostics for selective killing of bacteria targeted with gold nanoparticles. Biophys J.

[71] Norman RS, Stone JW, Gole A, Murphy CJ, Sabo-Attwood TL (2008). Targeted photothermal lysis of the pathogenic bacteria, *Pseudomonas aeruginosa*, with gold nanorods. Nano Lett.

[72] Wang S, Singh AK, Senapati D, Neely A, Yu H, Ra PC (2010). Rapid colorimetric identification and targeted photothermal lysis of salmonella bacteria by using bioconjugated oval-shaped gold nanoparticles. Chem -Eur J.

[73] Sun Z, Zhang Y, Yu H, Yan C, Liu Y, Hong S (2018). New solvent-stabilized few-layer black phosphorus for antibacterial applications. Nanoscale.

[74] Jo W, Kim MJ (2013). Influence of the photothermal effect of a gold nanorod cluster on biofilm disinfection. Nanotechnology.

[75] Castillo-Martínez JC, Martínez-Castañón GA, Martínez-Gutierrez F, Zavala-Alonso NV, Patiño-Marín N, Niño-Martinez N (2015). Antibacterial and antibiofilm activities of the photothermal therapy using gold nanorods against seven different bacterial strains. J Nanomater.

[76] He S, Zhu G, Sun Z, Wang J, Hui P, Zhao P (2020). 2D AuPd alloy nanosheets: one-step synthesis as imaging-guided photonic nano-antibiotics. Nanoscale Adv.

[77] Khantamat O, Li C, Yu F, Jamison AC, Shih W, Cai C (2015). Gold nanoshell-decorated silicone surfaces for the near-infrared (NIR) photothermal destruction of the pathogenic bacterium *E. faecalis*. ACS Appl Mater Interfaces.

[78] Pallavicini P, Donà A, Taglietti A, Minzioni P, Patrini M, Dacarro G (2014). Self-assembled monolayers of gold nanostars: a convenient tool for Near-IR photothermal biofilm eradication. Chem Commun.

[79] Mohamed MAA, Raeesi V, Turner PV, Rebbapragada A, Banks K, Chan WCW (2016). A versatile plasmonic thermogel for disinfection of antimicrobial resistant bacteria. Biomaterials.

[80] Ding X, Yuan P, Gao N, Zhu H, Yang YY, Xu Q (2017). Au-Ag core-shell nanoparticles for simultaneous bacterial imaging and synergistic antibacterial activity. Nanomedicine.

[81] Hu B, Wang N, Han L, Chen M, Wang J (2015). Core-shell-shell nanorods for controlled release of silver that can serve as a nanoheater for photothermal treatment on bacteria. Acta Biomater.

[82] Hu X, Zhao Y, Hu Z, Saran A, Hou S, Wen T (2013). Gold nanorods core/AgPt alloy nanodots shell: A novel potent antibacterial nanostructure. Nano Res.

[83] Peng Y, Liu Y, Lu X, Wang S, Chen M, Huang W (2018). Ag-Hybridized plasmonic Au-triangular nanoplates: highly sensitive photoacoustic/raman evaluation and improved antibacterial/photothermal combination therapy. J Mater Chem B.

[84] Hu D, Li H, Wang B, Ye Z, Lei W, Jia F (2017). Surface-adaptive gold nanoparticles with effective adherence and enhanced photothermal ablation of methicillin-resistant Staphylococcus aureus biofilm. ACS Nano.

[85] Ocsoy I, Yusufbeyoglu S, Yılmaz V, McLamore ES, Ildız N, Ülgen A (2017). DNA aptamer functionalized gold nanostructures for molecular recognition and photothermal inactivation of methicillin-resistant *Staphylococcus aureus*. Colloids Surf B.

[86] Hu B, Zhang L, Chen X, Wang J (2013). Gold nanorod-covered kanamycin-loaded hollow SiO_2_ (HSKAu(rod)) nanocapsules for drug delivery and photothermal therapy on bacteria. Nanoscale.

[87] Yang N, Wang C, Wang X, Li L (2018). Synthesis of photothermal nanocomposites and their application to antibacterial assays. Nanotechnology.

[88] Moon K, Bae J, Jin S, Oh S (2014). Infrared-mediated drug elution activity of gold nanorod-grafted TiO_2_ nanotubes. J Nanomater.

[89] Liu S, Zeng TH, Hofmann M, Burcombe E, Wei J, Jiang R (2011). Antibacterial activity of graphite, graphite oxide, graphene oxide, and reduced graphene oxide: membrane and oxidative stress. ACS Nano.

[90] Huang J, Zhou J, Zhuang J, Gao H, Huang D, Wang L (2017). Strong NIR-infrared absorbing and biocompatible CuS nanoparticles for rapid and efficient photothermal ablation of gram-positive and -negative bacteria. ACS Appl Mater Interfaces.

[91] D'Agostino A, Taglietti A, Desando R, Bini M, Patrini M, Dacarro G (2017). Bulk surfaces coated with triangular silver nanoplates: antibacterial action based on silver release and photo-thermal effect. Nanomaterials.

[92] Wang C, Irudayaraj J (2010). Multifunctional magnetic-optical nanoparticle probes for simultaneous detection, separation, and thermal ablation of multiple pathogens. Small.

[93] Yu T, Li P, Tseng T, Chen Y (2011). Multifunctional Fe₃O₄/alumina core/shell MNPs as photothermal agents for targeted hyperthermia of nosocomial and antibiotic-resistant bacteria. Nanomedicine.

[94] Jin Y, Deng J, Yu J, Yang C, Tong M, Hou Y (2015). Fe_5_C_2_ nanoparticles: a reusable bactericidal material with photothermal effects under near-infrared irradiation. J Mater Chem B.

[95] Hu X, Li L, Lu Y, Liu C, Lei Y, Zhang C (2017). Multifunctional CuS nanocrystals for inhibiting both osteosarcoma proliferation and bacterial infection by photothermal therapy. J Nanopart Res.

[96] Huang J, Zhou J, Zhuang J, Gao H, Huang D, Wang L (2017). Strong near-infrared absorbing and biocompatible CuS nanoparticles for rapid and efficient photothermal ablation of gram-positive and -negative bacteria. ACS Appl Mater Interfaces.

[97] Zhao Y, Cai Q, Qi W, Jia Y, Xiong T, Fan Z (2018). BSA-CuS nanoparticles for photothermal therapy of diabetic wound infection *in vivo*. ChemistrySelect.

[98] Qiao Y, Ping Y, Zhang H, Zhou B, Liu F, Yu Y (2019). Laser-activatable CuS nanodots to treat multidrug-resistant bacteria and release copper ion to accelerate healing of infected chronic nonhealing wounds. ACS Appl Mater Interfaces.

[99] Gao DY, Ji X, Wang JL, Wang YT, Li DL, Liu YB (2018). Engineering a protein-based nanoplatform as an antibacterial agent for light activated dual-modal photothermal and photodynamic therapy of infection in both the NIR I and II windows. J Mater Chem B.

[100] Suo H, Zhao X, Zhang Z, Wu Y, Guo C (2018). Upconverting LuVO4:Nd^3+^/Yb^3+^/Er^3+^@SiO_2_@Cu_2_S hollow nanoplatforms for self-monitored photothermal ablation. ACS Appl Mater Interfaces.

[101] Yin W, Yu J, Lv F, Yan L, Zheng LR, Gu Z (2016). Functionalized nano-MoS_2_ with peroxidase catalytic and near-infrared photothermal activities for safe and synergetic wound antibacterial applications. ACS Nano.

[102] Zhang W, Shi S, Wang Y, Yu S, Zhu W, Zhang X (2016). Versatile molybdenum disulfide based antibacterial composites for *in vitro* enhanced sterilization and *in vivo* focal infection therapy. Nanoscale.

[103] Gao Q, Zhang X, Yin W, Ma D, Xie C, Zheng L (2018). Functionalized MoS_2_ Nanovehicle with near-infrared laser-mediated nitric oxide release and photothermal activities for advanced bacteria-infected wound therapy. Small.

[104] Zhang C, Hu D, Xu J, Ma M, Xing H, Yao K (2018). Polyphenol-assisted exfoliation of transition metal dichalcogenides into nanosheets as photothermal nanocarriers for enhanced antibiofilm activity. ACS Nano.

[105] Yin Q, Tan L, Lang Q, Ke X, Bai L, Guo K (2018). Plasmonic molybdenum oxide nanosheets supported silver nanocubes for enhanced near-infrared antibacterial activity: Synergism of photothermal effect, silver release and photocatalytic reactions. Appl Catal B.

[106] Kim YK, Kang EB, Kim SM, Park CP, In I, Park SY (2017). Performance of NIR-mediated antibacterial continuous flow microreactors prepared by mussel-inspired immobilization of Cs_0.33_WO_3_ photothermal agents. ACS Appl Mater Interfaces.

[107] Liu Z, Liu J, Wang R, Du Y, Ren J, Qu X (2015). An efficient nano-based theranostic system for multi-modal imaging-guided photothermal sterilization in gastrointestinal tract. Biomaterials.

[108] Lai B, Chen D (2013). Vancomycin-modified LaB_6_@SiO_2_/Fe_3_O_4_ composite nanoparticles for near-infrared photothermal ablation of bacteria. Acta Biomater.

[109] Wang S, Singh AK, Senapati D, Neely A, Yu H, Ray PC (2010). Rapid colorimetric identification and targeted photothermal lysis of salmonella bacteria by using bioconjugated oval-shaped gold nanoparticles. Chem -Eur J.

[110] Lin D, Qin T, Wang Y, Sun X, Chen L (2014). Graphene oxide wrapped SERS tags: multifunctional platforms toward optical labeling, photothermal ablation of bacteria, and the monitoring of killing effect. ACS Appl Mater Interfaces.

[111] Feng Y, Chen Q, Yin Q, Pan G, Tu Z, Liu L (2019). Reduced graphene oxide functionalized with gold nanostar nanocomposites for synergistically killing bacteria through intrinsic antimicrobial activity and photothermal ablation. ACS Appl Bio Mater.

[112] Luoa J, Deng W, Yang F, Wu Z, Huang M, Gu M (2018). Gold nanoparticles decorated graphene oxide/nanocellulose paper for NIR laser-induced photothermal ablation of pathogenic bacteria. Carbohydr Polym.

[113] Wu P, Chen H, Chen S, Wang W, Yang K, Huang C Graphene oxide conjugated with polymers: a study of culture condition to determine whether a bacterial growth stimulant or an antimicrobial agent? J Nanobiotechnol. 018; 16: 1-20.

[114] Hea D, Yang T, Qian W, Qi C, Mao L, Yu X (2018). Combined photothermal and antibiotic therapy for bacterial infection via acidity-sensitive nanocarriers with enhanced antimicrobial performance. Appl Mater Today.

[115] Meeker DG, Jenkins SV, Miller EK, Beenken KE, Loughran AJ, Powless A (2016). Synergistic photothermal and antibiotic killing of biofilm-associated *Staphylococcus aureus* using targeted antibiotic-loaded gold nanoconstructs. ACS Infect Dis.

[116] Xu X, Liu X, Tan L, Cui Z, Yang X, Zhu S (2018). Controlled-temperature photothermal and oxidative bacteria killing and acceleration of wound healing by polydopamine-assisted Au-hydroxyapatite nanorods. Acta Biomater.

[117] Dai X, Zhao Y, Yu Y, Chen X, Wei X, Zhang X (2017). Single continuous near-infrared laser-triggered photodynamic and photothermal ablation of antibiotic-resistant bacteria using effective targeted copper sulfide nanoclusters. ACS Appl Mater Interfaces.

[118] Tian T, Shi X, Cheng L, Luo Y, Dong Z, Gong H (2014). Graphene-based nanocomposite as an effective, multifunctional, and recyclable antibacterial agent. ACS Appl Mater Interfaces.

[119] Dai X, Zhao Y, Yu Y, Chen X, Wei X, Zhang X (2018). All-in-one NIR-activated nanoplatforms for enhanced bacterial biofilm eradication. Nanoscale.

[120] Yang F, Feng Y, Fan X, Zhang M, Wang C, Zhao W (2019). Biocompatible graphene-based nanoagent with NIR and magnetism dual-responses for effective bacterial killing and removal. Colloids Surf B.

[121] Liu R, Wang X, Ye J, Xue X, Zhang F, Zhang H (2018). Enhanced antibacterial activity of silver-decorated sandwich-like mesoporous silica/reduced graphene oxide nanosheets through photothermal effect. Nanotechnology.

[122] Wang N, Hu B, Chen M, Wang J (2015). Polyethylenimine mediated silver nanoparticle-decorated magnetic graphene as a promising photothermal antibacterial agent. Nanotechnology.

[123] Hu B, Wang N, Han L, Chen M, Wang J (2015). Magnetic nanohybrids loaded with bimetal core-shell-shell nanorods for bacteria capture, separation, and near-infrared photothermal treatment. Chem -Eur J.

[124] Ko YC, Fang HY, Chen DH (2017). Fabrication of Ag/ZnO/reduced graphene oxide nanocomposite for SERS detection and multiway killing of bacteria. J Alloys Compd.

[125] Han L, Che S (2018). An overview of materials with triply periodic minimal surfaces and related geometry: from biological structures to self-assembled systems. Adv Funct Mater.

[126] Qin L, Jiang S, He H, Ling G, Zhang P (2020). Functional black phosphorus nanosheets for cancer therapy. J Controlled Release.

[127] Zhang D, Liu HM, Shu X, Feng J, Yang P, Dong P (2020). Nanocopper-loaded Black phosphorus nanocomposites for efficient synergistic antibacterial application. J Hazard Mater.

[128] Liang M, Zhang Mi, Yu S, Wu Q, Ma K, Chen Y (2020). Silver-laden black phosphorus nanosheets for an efficient *in vivo* antimicrobial application. Small.

[129] Aksoy İ, Küçükkeçeci H, Sevgi F, Metin Ö, Patir IH (2020). Photothermal antibacterial and antibiofilm activity of black phosphorus/gold nanocomposites against pathogenic bacteria. ACS Appl Mater Interfaces.

[130] Saleem J, Wang L, Chen C (2018). Carbon-based nanomaterials for cancer therapy *via* targeting tumor microenvironment. Adv Healthcare Mater.

[131] Wang X, Shao J, Raouf MAE, Xie H, Huang H, Wang H (2018). Near-infrared light-triggered drug delivery system based on black phosphorus for *in vivo* bone regeneration. Biomaterials.

[132] Wang Y, Hu X, Zhang L, Zhu C, Wang J, Li Y (2019). Bioinspired extracellular vesicles embedded with black phosphorus for molecular recognition-guided biomineralization. Nat Commun.

[133] Cao Y, Dong H, Yang Z, Zhong X, Chen Y, Dai W (2017). Aptamer-conjugated graphene quantum dots/porphyrin derivative theranostic agent for intracellular cancer-related MicroRNA detection and fluorescence-guided photothermal/photodynamic synergetic therapy. ACS Appl Mater Interfaces.

[134] Ge J, Lan M, Zhou B, Liu W, Guo L, Wang H (2014). A graphene quantum dot photodynamic therapy agent with high singlet oxygen generation. Nat Commun.

[135] Mei L, Gao X, Shi Y, Cheng C, Shi Z, Jiao M (2020). Augmented graphene quantum dot-light irradiation therapy for bacteria-infected wounds. ACS Appl Mater Interfaces.

[136] Ge J, Jia Q, Liu W, Lan M, Zhou B, Guo L (2016). Carbon dots with intrinsic theranostic properties for bioimaging, red-light-triggered photodynamic/photothermal simultaneous therapy *in vitro* and *in vivo*. Adv Healthcare Mater.

[137] Nie X, Jiang C, Wu S, Chen W, Lv P, Wang Q (2020). Carbon quantum dots: A bright future as photosensitizers for *in vitro* antibacterial photodynamic inactivation. J Photochem Photobiol B.

[138] Kim H, Mun S, Choi Y (2013). Photosensitizer-conjugated polymeric nanoparticles for redox-responsive fluorescence imaging and photodynamic therapy. J Mater Chem B.

[139] Lu C, Sun F, Liu Y, Xiao Y, Qiu Y, Mu H (2019). Versatile chlorin e6-based magnetic polydopamine nanoparticles for effectively capturing and killing MRSA. Carbohydr Polym.

[140] Li C, Lin F, Sun W, Wu F, Yang H, Lv R (2018). Self-assembled rose bengal-exopolysaccharide nanoparticles for improved photodynamic inactivation of bacteria by enhancing singlet oxygen generation directly in the solution. ACS Appl Mater Interfaces.

[141] Liu M, He D, Yang T, Liu W, Mao L, Zhu Y (2018). An efficient antimicrobial depot for infectious site-targeted chemo-photothermal therapy. J Nanobiotechnol.

[142] Ethirajan M, Chen Y, Joshi P, Pandey RK (2011). The role of porphyrin chemistry in tumor imaging and photodynamic therapy. Chem Soc Rev.

[143] Park W, Bae B, Na K (2016). A highly tumor-specific light-triggerable drug carrier responds to hypoxic tumor conditions for effective tumor treatment. Biomaterials.

[144] Tüzün Y, Wolf R, Engin B, Keçici AS, Kutlubay Z (2015). Bacterial infections of the folds (intertriginous areas). Clin Dermatol.

[145] Lee E, Li X, Oh J, Kwon N, Kim G, Kim (2020). A boronic acid-functionalized phthalocyanine with an aggregation-enhanced photodynamic effect for combating antibiotic-resistant bacteria. Chem Sci.

[146] Kim SH, Kang EB, Jeong CJ, Sharker SM, In I, Park SY (2015). Light controllable surface coating for effective photothermal killing of bacteria. ACS Appl Mater Interfaces.

[147] Korupallia C, Huanga C, Lin W, Pan W, Lin P, Wan W (2017). Acidity-triggered charge-convertible nanoparticles that can cause bacterium-specific aggregation *in situ* to enhance photothermal ablation of focal infection. Biomaterials.

[148] Feng Y, Liu L, Zhang J, Aslan H, Dong M (2017). Photoactive antimicrobial nanomaterials. J Mater Chem B.

[149] Chen Y, Gao Y, Chen Y, Liu L, Mo A, Peng Q (2020). Nanomaterials-based photothermal therapy and its potentials in antibacterial treatment. J Controlled Release.

[150] Tan L, Li J, Liu X, Cui Z, Yang X, Zhu S (2018). Rapid biofilm eradication on bone implants using red phosphorus and near-infrared light. Adv Mater.

[151] Yang X, Li Z, Ju E, Ren J, Qu X (2014). Reduced graphene oxide functionalized with a luminescent rare-earth complex for the tracking and photothermal killing of drug-resistant bacteria. Chem - Eur J.

[152] Laport MS, Silva da MR, Silva CC, Bastos MF, Giambiagi-deMarval M (2003). Heat-resistance and heat-shock response in the nosocomial pathogen *Enterococcus faecium*. Curr Microbiol.

[153] Sofía M, Martin FD, Paolo NC, Martin GB (2015). Antimicrobial surfaces from incorporated nano-agents. Curr Bionanotechnol. (Discontinued).

[154] Kwon EJ, Skalak M, Bertucci A, Braun G, Ricci F, Ruoslahti E (2017). Porous silicon nanoparticle delivery of tandem peptide anti-infectives for the treatment of pseudomonas aeruginosa lung infections. Adv Mater.

[155] Zhang CY, Lin W, Gao J, Shi X, Davaritouchaee M, Nielsen AE (2019). pH-Responsive nanoparticles targeted to lungs for improved therapy of acute lung inflammation/injury. ACS Appl Mater Interfaces.

[156] Yang Y, Ding Y, Fan B, Wang Y, Mao Z, Wang W (2020). Inflammation-targeting polymeric nanoparticles deliver sparfloxacin and tacrolimus for combating acute lung sepsis. J Controlled Release.

[157] Gao Y, Wang J, Chai M, Li X, Deng Y, Jin Q (2020). Size and charge adaptive clustered nanoparticles targeting the biofilm microenvironment for chronic lung infection management. ACS Nano.

[158] Chiang W, Lin T, Sureshbabu R, Chia W, Hsiao H, Liu H (2015). A rapid drug release system with a NIR light-activated molecular switch for dual-modality photothermal/antibiotic treatments of subcutaneous abscesses. J Controlled Release.

[159] Ji H, Dong K, Yan Z, Ding C, Chen Z, Ren J (2016). Bacterial hyaluronidase self-triggered prodrug release for chemo-photothermal synergistic treatment of bacterial infection. Small.

[160] Ga G, Jiang Y, Jia H, Wu F (2019). Near-infrared light-controllable on-demand antibiotics release using thermo-sensitive hydrogel-based drug reservoir for combating bacterial infection. Biomaterials.

[161] Tang Z, Liu Y, He M, Bu W (2018). Chemodynamic therapy: tumour microenvironment-mediated fenton and fenton-like reactions. Angew Chem Int Ed.

[162] Lin L, Song J, Song L, Ke K, Liu Y, Zhou Z (2018). Simultaneous fenton-like ion delivery and glutathione depletion by MnO_2_-based nanoagent to enhance chemodynamic therapy. Angew Chem Int Ed.

[163] Hyslop PA, Hinshaw DB, Scraufstatter IU, Cochrane CG, Kunz S, Vosbeck K Hydrogen peroxide as a potent bacteriostatic antibiotic: Implications for host defense, Free Radicals Biol Med. 1995; 19: 31-37.

[164] Naha PC, Liu Y, Hwang G, Huang Y, Gubara S, Jonnakuti V (2019). Dextran-coated iron oxide nanoparticles as biomimetic catalysts for localized and pH-activated biofilm disruption. ACS Nano.

[165] Xu B, Wang H, Wang W, Gao L, Li S, Pan X (2019). A single-atom nanozyme for wound disinfection applications. Angew Chem Int Ed.

[166] Wang Z, Dong K, Liu Z, Zhang Y, Chen Z, Sun H (2017). Activation of biologically relevant levels of reactive oxygen species by Au/g-C_3_N_4_ hybrid nanozyme for bacteria killing and wound disinfection. Biomaterials.

[167] Fang G, Li W, Shen X, Perez-Aguilar JM, Chong Y, Gao X (2018). Differential Pd-nanocrystal facets demonstrate distinct antibacterial activity against Gram-positive and Gram-negative bacteria. Nat Commun.

[168] Sun D, Pang X, Cheng Y, Ming J, Xiang S, Zhang C (2020). Ultrasound-switchable nanozyme augments sonodynamic therapy against multidrug-resistant bacterial infection. ACS Nano.

[169] Zhao X, Jia Y, Li J, Dong R, Zhang J, Ma C (2018). Indole derivative-capped gold nanoparticles as an effective bactericide *in vivo*. ACS Appl Mater Interfaces.

[170] Cai S, Jia X, Han Q, Yan X, Yang R, Wang C (2017). Porous Pt/Ag nanoparticles with excellent multifunctional enzyme mimic activities and antibacterial effects. Nano Res.

[171] Zhang Y, Sun P, Zhang L, Wang Z, Wang F, Dong K (2019). Silver-infused porphyrinic metal-organic framework: surface-adaptive, on-demand nanoplatform for synergistic bacteria killing and wound disinfection. Adv Funct Mater.

[172] Shen Z, Liu T, Li Y, Lau J, Yang Z, Fan W (2018). Fenton-reaction-acceleratable magnetic nanoparticles for ferroptosis therapy of orthotopic brain tumors. ACS Nano.

[173] Rizzelloa L, Pompa PP (2014). Nanosilver-based antibacterial drugs and devices: Mechanisms, methodological drawbacks, and guidelines. Chem Soc Rev.

[174] Zhao Y, Guo Q, Dai X, Wei X, Yu Y, Chen X (2018). A biomimetic non-antibiotic approach to eradicate drug-resistant infections. Adv Mater.

[175] Pelgrift RY, Friedman AJ (2013). Nanotechnology as a therapeutic tool to combat microbial resistance. Adv Drug Deliv Rev.

[176] Yang G, Zeng S, Phua F, Bindra AK, Zhao Y (2019). Degradability and clearance of inorganic nanoparticles for biomedical applications. Adv Mater.

[177] Chen Y, Ye D, Wu M, Chen H, Zhang L, Shi J (2014). Break-up of two-dimensional MnO_2_ nanosheets promotes ultrasensitive pH-triggered theranostics of cancer. Adv Mater.

[178] Yu L, Hu P, Chen Y (2018). Gas-generating nanoplatforms: material chemistry, multifunctionality, and gas therapy. Adv Mater.

[179] Li G, Yu S, Xue W, Ma D, Zhang W (2018). Chitosan-graft-PAMAM loading nitric oxide for efficient antibacterial application. Chem-Eng J.

[180] Gao Q, Zhang X, Yin W, Ma D, Xie C, Zheng L (2018). Functionalized MoS_2_ Nanovehicle with near-infrared laser-mediated nitric oxide release and photothermal activities for advanced bacteria-infected wound therapy. Small.

[181] Jin Z, Wen Y, Hu Y, Chen W, Zheng X, Guo W (2017). MRI-guided and ultrasound-triggered release of NO by advanced nanomedicine. Nanoscale.

[182] Yang T, Zelikin AN, Chandrawati R (2018). Progress and promise of nitric oxide-releasing platforms. Adv Sci.

[183] Cejka C, Kubinova S, Cejkova J (2019). The preventive and therapeutic effects of molecular hydrogen in ocular diseases and injuries where oxidative stress is involved. Free Radical Res.

[184] Wan W, Lin Y, Chen H, Huang C, Shih P, Bow Y (2017). *In situ* nanoreactor for photosynthesizing H_2_ gas to mitigate oxidative stress in tissue inflammation. J Am Chem Soc.

[185] Li H, Shen L, Ge J, Zhang R (2017). The transfer of hydrogen from inert gas to therapeutic gas. Med Gas Res.

[186] Yu S, Li G, Zhao P, Cheng Q, He Q, Ma D (2019). Near-laser-controlled hydrogen-releasing PdH nanohydride for synergistic hydrogen-photothermal antibacterial and wound-healing therapies. Adv Funct Mater.

[187] Nguyen D, Nguyen TK, Rice SA, Boyer C (2015). CO-Releasing polymers exert antimicrobial activity. Biomacromolecules.

[188] Bang CS, Kruse R, Johansson K, Persson K (2016). Carbon monoxide releasing molecule-2 (CORM-2) inhibits growth of multidrug resistant uropathogenic *Escherichia coli* in biofilm and following host cell colonization. BMC Microbiol.

[189] Ward JS, Lynam JM, Moir J, Fairlamb JS (2014). Visible-light-induced CO release from a therapeutically viable tryptophan-derived manganese(I) carbonyl (TryptoCORM) exhibiting potent inhibition against *E. coli*. Chem - Eur J.

[190] Murray TS, Okegbe C, Gao Y, Kazmierczak BI, Motterlini R, Dietrich LEP (2012). The carbon monoxide releasing molecule CORM-2 attenuates pseudomonas aeruginosa biofilm formation. PLoS One.

[191] Ma W, Chen X, Fu L, Zhu J, Fan M, Chen J Ultra-efficient antibacterial system based on photodynamic therapy and CO gas therapy for synergistic antibacterial and ablation biofilms, ACS Appl Mater Interfaces. 2020; 12: 22479-22491.

[192] Qiao Y, He J, Chen W, Yu Y, Li W, Du Z (2020). Light-activatable synergistic therapy of drug-resistant bacteria-infected cutaneous chronic wounds and nonhealing keratitis by cupriferous hollow nanoshells. ACS Nano.

[193] Drewry AM, Hotchkiss RS (2015). Revising definitions of sepsis. Nat Rev Nephrol.

[194] Wu M, Qi G, Liu X, Duan Y, Liu J, Liu B (2020). Bio-orthogonal AIEgen for specific discrimination and elimination of bacterial pathogens via metabolic engineering. Chem Mater.

